# Context Definition and Query Language: Conceptual Specification, Implementation, and Evaluation

**DOI:** 10.3390/s19061478

**Published:** 2019-03-26

**Authors:** Alireza Hassani, Alexey Medvedev, Pari Delir Haghighi, Sea Ling, Arkady Zaslavsky, Prem Prakash Jayaraman

**Affiliations:** 1 Faculty of Information Technology, Monash University, Melbourne 3145, Australia; alexey.medvedev@monash.edu (A.M.); pari.delir.haghighi@monash.edu (P.D.H.); chris.ling@monash.edu (S.L.); 2 School of Information Technology, Deakin University, Geelong, Vic 3216, Australia; arkady.zaslavsky@deakin.edu.au; 3 Department of Computer Science and Software Engineering, Swinburne University of Technology, Melbourne 3122, Australia; pjayaraman@swin.edu.au

**Keywords:** context, query, language, IoT, CMP

## Abstract

As IoT grows at a staggering pace, the need for contextual intelligence is a fundamental and critical factor for IoT intelligence, efficiency, effectiveness, performance, and sustainability. As the standardisation efforts for IoT are fast progressing, efforts in standardising context management platforms led by the European Telecommunications Standards Institute (ETSI) are gaining more attention from both academic and industrial research organizations. These standardisation endeavours will enable intelligent interactions between ‘things’, where things could be devices, software components, web-services, or sensing/actuating systems. Therefore, having a generic platform to describe and query context is crucial for the future of IoT applications. In this paper, we propose Context Definition and Query Language (CDQL), an advanced approach that enables things to exchange, reuse and share context between each other. CDQL consists of two main parts, namely: context definition model, which is designed to describe situations and high-level context; and Context Query Language (CQL), which is a powerful and flexible query language to express contextual information requirements without considering details of the underlying data structures. An important feature of the proposed query language is its ability to query entities in IoT environments based on their situation in a fully dynamic manner where users can define situations and context entities as part of the query. We exemplify the usage of CDQL on three different smart city use cases to highlight how CDQL can be utilised to deliver contextual information to IoT applications. Performance evaluation has demonstrated scalability and efficiency of CDQL in handling a fairly large number of concurrent context queries.

## 1. Introduction

During the last decade, context-awareness in the Internet of Things (IoT) environment gained much attention from researchers and industry. As IoT evolves, the need for accessing contextual information in real time becomes a crucial factor for the improvement of IoT applications. According to the widely accepted definition of context proposed by Dey, context is “any information that can be used to characterize the situation of an entity” [1]. Since the early 1990s, a large body of research has been conducted on context/context-awareness in pervasive computing to enable intelligent adaptation of applications allowing them to perform their tasks in an efficient, proactive, and autonomous manner [2], according to the context of its users or other involved entities.

IoT entities, which include sensors, mobile devices, connected cars, smart meters, and other smart devices are rich sources of data that is fundamental for reasoning about context of users, applications, and environment. In most cases, IoT-based smart services and applications are responsible for converting raw data coming from IoT data sources to high-level context. However, most of these applications and services are designed to provide context within closed loop systems (silos). They do not provide standard mechanisms or approaches to discover, share, and distribute context across multiple IoT applications and services, especially when these services are developed and operated by different organisations/vendors. In other words, if context generated by one IoT device is required by another IoT application, current systems cannot share this context without manual integration. A critical factor that will underpin the success of future IoT applications and services in order to provide more significant benefits to customers is the ability of applications and devices (machines) to exchange context seamlessly.

A promising solution to address the problem mentioned above is to build a middleware platform that manages interaction with sources of context and offers contextual information to context-aware applications as a service. A notable number of context management approaches have been proposed; surveys of which have been published for instance in [3,4,5,6]. Existing context management systems can be classified into three main generations. The earliest generation, such as the Active Badge System [7] only focused on utilising location data. The second generation comprised such systems as Context Toolkit [1], SOCAM [8], and Cobra [9]. These systems tried to achieve a higher level of generality and supporting more types of context. However, these systems suffer from a number of common constraints that makes them inefficient to be used in real world context-aware systems. These constraints include lack of fault tolerance and scalability, poor interoperability support and naïve reasoning just to name a few, which lead to low market penetration of these systems. The effort of the research community to address these limitations lead to the development of third generation context middleware platforms, such as CA4IoT [10] and CAMPUS [11]. While they successfully addressed some of the mentioned limitations, they failed to evolve into an industry standard level.

We believe the main shortcoming of these middleware systems is the lack of a comprehensive and flexible context query language (CQL) that allows context-aware applications to repurpose existing contextual data based on their specific requirements. As a result, in this paper, in continuation of our efforts to develop a standard query language for sharing and exchanging context [12], we propose a comprehensive, and flexible Context Definition and Query Language (CDQL). CDQL provides a generic and flexible approach to defining, representing, inferring, monitoring, and querying context in IoT applications.

CDQL is one of the major building blocks of the Context-as-a-Service (CoaaS) platform [13]. CoaaS is a context management middleware which is responsible for facilitating sharing context provided by different sources with context-aware applications. The CoaaS enables global standardization and interworking among different context providers and consumers in IoT environments. Context as a Service (CoaaS) forms an important part of the service offerings in the EU Horizon-2020 project called bIoTope (www.biotope-h2020.eu)—Building IoT OPen Innovation Ecosystem for connected smart objects.

The main contributions of this paper are summarised below:

- analysing the existing Context Query Languages proposed in the literature and deriving a refined set of functional requirements for CQL.
- proposing a refined version of CDQL and presenting a formal specification of its syntax using Extended Backus-Naur Form (EBNF) statements.
- demonstrating the feasibility and applicability of CDQL by presenting exemplary queries for each of the use cases discussed in the paper.
- conducting multiple experiments based on real-world and synthetic dataset to evaluate the performance of an implementation of the proposed language in the CoaaS platform.

This paper is organised as follows: Section 2 provides three IoT enabled smart city use cases. Section 3 summarises the main research directions the related work in the area. Section 4 describes the high-level architecture of the CoaaS platform. It also sets the main terminology and definitions. Section 5 presents the proposed Context Definition and Query Language, and Section 6 presents the proof of concept testing and performance evaluation. Section 7 concludes the paper and sets directions for future work.

## 2. Motivating Use Cases

In this section, we present three different motivating use cases based on smart city scenarios that highlight the need for a context management framework with a flexible, dynamic, and easy to use approach to define, advertise, discover/acquire, and query context in IoT environment.

### 2.1. Use Case 1: School Safety

**Figure 1:**
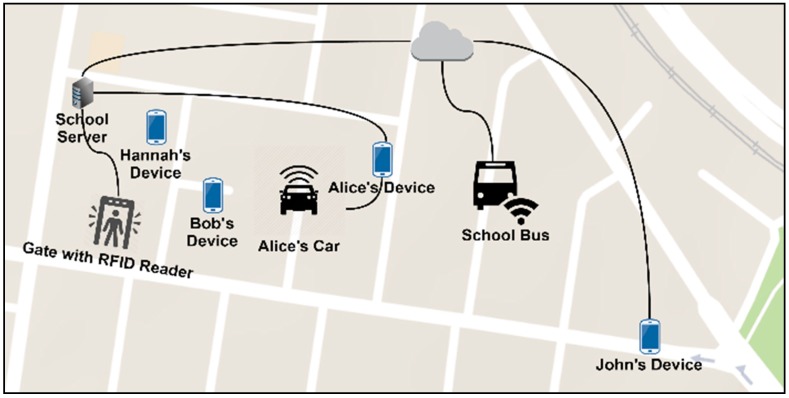
School safety use-case.

The first use case under consideration, which is called school safety, is depicted in Figure 1. In this use case, a user called John wants to pick-up his daughter, Hannah, from school. On his way to school, due to unexpected traffic, he realises that he cannot arrive at the school on time. Realising this, a smart IoT system begins to determine alternatives to achieve the goal “pickup Hannah”. An option could be to request another trusted parent to pick-up Hannah from school on John’s behalf. In order to represent this context request, several factors should be considered, namely:

- The selected parent(s) for picking up Hannah should be trusted by John;
- The selected parent(s) should have a car with an extra seat for Hanna;
- The selected parent(s) should be close enough to the school;
- The child of the selected parent(s) should finish the school at the same time as Hannah;
- The child of the selected parent(s) should be currently at school.

Additionally, this process needs to be automated, so John’s device can automatically trigger the same query, “pickup Hannah” whenever he is running late.

### 2.2. Use Case 2: Smart Parking Recommender

The second use case we consider in this paper focuses on facilitating the development of a context-aware IoT application that suggests parking facilities to drivers. Such an application needs to (1) have access to live data regarding availability of different parking facilities owned by different providers (e.g., city administrators, building owners, and organizations); (2) provide personalized recommendations to users, considering factors such as user preferences, car specifications, and related environmental conditions such as weather; and (3) continuously monitor relevant context and notify the driver about any changes in situations that can affect his/her experience, e.g., notify the driver if the suggested parking becomes unavailable or another parking with better conditions (such as cheaper or closer to the destination) becomes available.

### 2.3. Use Case 3: Vehicle Pre-Conditioning

The third use case under consideration is a smart connected electric vehicle pre-conditioning use-case. Pre-conditioning allows the drivers to begin their journey with a properly heated or cooled cabin. The pre-conditioning use case requires continuous monitoring of several situations (computed from context of various IoT smart things and applications) such as the car’s location (provided by the connected car), the driver’s location, the driver’s calendar (provided by the driver’s smart mobile device), and weather conditions (obtained from nearby IoT weather stations) to name a few. Moreover, such a use case also requires specific reasoning to infer the likelihood of the driver commencing a journey, e.g., walking past the car vs. walking to the car to begin a journey. Finally, based on inferred situations, an actuation signal to start the pre-conditioning process will need to be sent to the car’s onboard computer.

Based on these use cases and the considerations above, we have identified six main requirements for a context query language:

1. Support for complex context queries concerning various contexts entities and constraints (e.g., join queries);
2. Support for domain-based standards (e.g., ontologies) and facilitate interoperability.
3. Support for both pull-based and push-based queries;
4. Support for aggregating and reasoning functions to query high-level context and also mitigate the privacy issues of sharing sensitive providers’ data with external consumers;
5. Support for continuous and situation/event-based queries;
6. Support for different aspects of context such as imperfectness, uncertainty, Quality of Context (QoC), and Cost of Context (CoC).

## 3. Related Works

### 3.1. Definition of Context and Context-Awareness

The term context (from Latin ‘contextus’, from ‘con-’ together + ‘texere’ to weave) is defined in Oxford dictionary as “The circumstances that form the setting for an event, statement, or idea, and in terms of which it can be fully understood”. While this definition is understandable for most people, it is not clear enough to be used as a formal definition. Therefore, a considerable number of attempts have been made by many researchers to develop a generic and standard definition for the term context. In this section, in order to find a formal definition that meets the requirements of this research, we will look at the existing context definitions used in the literature. These works can be classified into three main categories: defining context by example, defining context by synonyms, and conceptual definitions which is a formal approach and concentrates on the relationships and structure of contextual information [14].

The term context-aware was introduced for the first time by Theimer et al. [15]. They define context as “where you are, who you are with, and what resources are nearby”. In this definition, location is considered as the core element of the context. However, Theimer et al. partially include contextual information about nearby people and objects in their definition as well. Abowd et al. [16] also proposed a similar definition and identified the five ‘W’s (who, what, where, when, why) as the minimum information that is necessary to understand context. This type of definitions that describes context by example is hard to use as these definitions are limited to defining certain types of context and cannot be applied to wider categories of context.

Another sub-class of context definition describes context by simply providing synonyms for context, referring to context as the environment or situation [17,18,19,20,21,22]. Brown et al. [21] defined context as location, identity of nearby people, and time of the day. Ryan et al. [22] reported on fieldwork where they viewed context as location, environment, identity, and time. Franklin and Flaschbart [20] saw it as the situation of the user. Ward et al. [17] viewed context as the state of the application’s surroundings, and Rodden et. al. [19] defined it as the application’s setting. Hull et al. [18] included the entire environment by defining context to be aspects of the current situation. These definitions are more general than enumerations, but this generality is also a limitation. These definitions provide little guidance to analyse the constituent elements of context, much less identify them. Furthermore, these definitions are also inadequate to identify new context [1].

Some other researchers try to define context formally. Schmidt et al. [23] define context as “knowledge about the user’s and IT device’s state, including surroundings, situation, and to a less extent, location”. Another formal definition is provided by Chen and Kotz [24]. They defined context as “a set of environmental states and settings that either determines an application’s behaviour or in which an application event occurs and is interesting to the user.”

Another popular formal definition of context is proposed by Dey. He defines context as “any information that can be used to characterise the situation of an entity. An entity is a person, place, or object that is considered relevant to the interaction between a user and an application, including the user and applications themselves” [1]. We adopt this definition for our work.

As we mentioned earlier, the term context-aware was introduced for the first time by Theimer et al. [15]. Based on their definition, a software is context-aware if it “adapts according to the location of the user, the collection of the nearby people, hosts, and accessible devices, as well as to changes to such things over time.” [15]. Later, a similar definition was proposed by Ryan et al. [22].

Abowd et al. [25] showed that those definitions are too specific to be used as a yardstick to identify whether a given application is context-aware or not. Therefore, to solve this problem, Abowd et al. provide their definition of context awareness as follows: “A system is context-aware if it uses context to provide relevant information and/or services to the user, where relevancy depends on the user’s task” [25]. Later, Chen et al. defined context awareness as “a computer system’s ability to provide relevant services and information to users based their situational conditions” [26]. These two definitions, proposed by Abowd et al. and Chen et al., focus on the provisioning of information and services to the user.

However, some other researchers had another point of view and proposed a more general definition for context-awareness. For instance, in the work done by Razzaque et al. [27], context awareness was defined as “a term from computer science, which is used for devices that have information about the circumstances under which they operate and can react accordingly”. Becker et al. [28] stated that “an application is context-aware if it adapts its behaviour depending on the context”. Baldauf et al. introduced a new aspect of context-aware systems (i.e., self-adaptiveness) and defined it as a system that is “able to adapt their operations to the current context without explicit user intervention and thus aim at increasing usability and effectiveness by taking environmental context into account” [3]. Similarly, Huebscher et al. defined context-awareness as “the ability of an application to adapt itself to the context of its user(s)” [29]. The definitions by Baldauf et al. and Huebscher et al. highlight the context of the user.

In our view, both the context of the user of an application and the context of the application itself are essential; the following definition of context-awareness will be used in this paper: An application is context-aware if it aligns its behaviour based on context of any related entity to the application, including the user and application themselves.

### 3.2. Context Management and Provisioning

The management and provisioning of context information are essential elements for realising context-aware services and applications. In this sub-section, we review the main aspects and functionalities of context management platforms (CMP).

Knappmeyer et al. [6] subdivide the major functionalities of CMPs into six categories:

1. ‘Sensor Data Acquisition ‘deals with how raw information about any context is fetched and used as input to the middleware. It is vital that the system can cope with a variety of heterogeneous sources and sensors simultaneously. Sensors may be physical, virtual, or logical. Depending on the intelligence and computational power, pre-processing and filtering may be performed by the sensor nodes themselves or as part of the middleware functionality. Both synchronous and asynchronous sources are generally supported.
2. ‘Context Storage’ refers to the mechanism of persisting contextual information in the middleware. Having a proper context storage technique has two main benefits. Caching strategies allow for faster provisioning of the necessary context since repeated processing stages may be omitted. Moreover, the storage of expired context in a history database enables the analysis of previous situations. Such information can be used to determine habits and long-term intentions taking successive sequences of activities towards the desired goal into account.
3. ‘Context Lookup & Discovery’ provide means for an application, service or actuator to identify the available context and how to acquire and query for it. Commonly used approaches include lookup tables, semantic queries or legacy web service mechanisms such as SOAP (Simple Object Access Protocol) and WSDL (Web Services Description Language).
4. ‘Context Diffusion & Distribution’ are related to the output of a middleware system, i.e., how context information is made available to the consumers. This encompasses not only the definition of query models (e.g., key-value based, or SQL based) but also the mode of communication. Communication protocols may support event-driven asynchronous publish/subscribe mechanisms to notify the application layer about context changes of interest. Additionally, synchronous on-demand queries may be supported by the middleware.
5. ‘Privacy, Security, and Access Control’ are considered as vital tasks in context management middleware’s since they might expose users’ sensitive information to untrusted external systems**.**
6. ‘Context Processing & Reasoning’ refer to the capability of inferring context from raw sensor data or existing primitive low-level context. The middleware may apply feature extraction, description logic, rule-based reasoning or probabilistic inference on behalf of the application layer, hence saving battery consumption on mobile resource-constrained devices. A powerful middleware should support modularity so that numerous processing mechanisms and algorithms can be plugged in.

This paper will mainly focus on addressing the third and fourth functionalities, which are Context Lookup & Discovery and Context Diffusion & Distribution.

### 3.3. Semantic Web for Internet of Things

As mentioned in the previous section, one of the main functionates of a CMP is context lookup and discovery. A similar concept was raised and studied in the realm of Semantic Web Services (SWS) [30] to add automation and dynamics to traditional web services. SWS aims at providing formal descriptions of requests and web services that can be exploited to automate several tasks in the web services usage process, including dynamic discovery of services.

During the last two decades, a large body of research has been conducted on definition and composition of semantic service, especially in the domain of Semantic Web Service (SWS). These efforts led to the development of several web service description languages, such as Semantic Markup for Web Services (OWL-S) [31], Web Service Modelling Ontology (WSMO) [32], and Semantic Annotation for WSDL and XML Schema (SAWSDL) [33].

Most of the above-mentioned languages to some extent allow specifying services in terms of their signature (i.e., inputs and outputs of the service), behavioural specification (i.e., preconditions and effects), and the non-functional properties (NFPs). However, all of these languages suffer from the same limitation that makes them insufficient to describe IoT-services and their context-related aspects. To overcome these shortcomings, a number of different approaches have been proposed [34,35,36]. However, they do not fully support different types and aspects of context and lack an expressive language to represent them.

In this paper, we adopted OWL-S as the basic upper ontology for IoT-Service description by taking advantage of its flexibility and dynamicity in service composition.

### 3.4. Context Query Languages

In this section, we will review existing context query languages (CQL). Query languages are essential for querying context and determine the way context requests are represented. A wide range of query languages is employed for querying contextual information. Existing CQLs can be categorised into five sub-classes: SQL-based, RDF-based, XML-based, API-based, and graph-based CQLs. In the work done by Delir Haghighi et al. [37], an evaluation of different CQLs is presented. They compare different CQLs and demonstrate that SQL-based, XML-based, and RDF-based CQLs are more effective and powerful compared to the other subclasses.

Therefore, in the rest of this section, we provide a critical review and comparison of existing context query languages that fall into these three subclasses of context query languages (SQL-based, XML-based, and RDF-based CQLs). Furthermore, in order to accurately identify to what extent existing CQLs can support the needed requirements for CoaaS, we try to illustrate the applicability of each approach to be used as a context query language in CoaaS by considering the aforementioned school scenario.

SQL is the most popular declarative query language which is designed for accessing data from traditional databases. However, directly utilising SQL is not possible as context data has its characteristics that are different from relational database data. Compared to traditional database data, context data has its own special characteristics. According to [37], context:

- Can be dynamic or static.
- Can be continuous data streams.
- Can be temporal, erroneous, ambiguous, unavailable or incomplete.
- Can be spatial.
- Can be unstructured.
- Can be a situation that is derived and reasoned from other context.

Considering the school safety scenario, it is not possible to implement such a complex query by only using native SQL. Therefore, some researchers extend traditional SQL by adding optional instructions to support querying on context data.

Riva et al. [38] proposed a SQL-based CQL to provide contextual information for mobile applications called Contory. The proposed context query language consists of three mandatory clauses, namely SELECT, FROM, and WHERE. The SELECT clause identifies the type of the required context item (e.g., location, light, temperature, and activity). FROM clause specifies type and characteristics of the sources from which desired context data should be collected. Lastly, the WHERE clause filters context values according to specific requirements on their associated context metadata. Furthermore, they defined four optional clauses to provide a better filtering functionality and support event-based queries. The first clause is ‘freshness’ which identifies how recent the context data must be. On the other hand, the other three clauses (i.e., DURATION, EVERY, and EVENT) are responsible for supporting event-based and continuous/periodic queries.

The main shortcoming of Contory that makes it inappropriate to be used as the main interface of a CMP is its simplified data model, which does not have a mechanism to indicate the entity of interest in a query. For example, while it allows querying temperature, it does not support querying temperature of a specific oven. Furthermore, another shortcoming of this approach is the lack of supporting context processing operations. On top of these, Contory is not interoperable with different external infrastructures and sensor devices. Last but not least, this language does not support querying multiple sources of context simultaneously (in one query).

Another SQL-based query language is proposed by Henricksen et al. [39]. They developed a context management system on top of the Context Modelling Language (CML). CML is a powerful modelling approach for describing information’s type, their classification, and quality of context. In their proposed system, a simple API is designed for accessing the context information. The context management framework for CML [39] extends ORM to map its models to relational data schemes. Therefore, SQL can be used to retrieve contextual information. In other words, context queries are internally mapped to SQL [40]. While SQL supports some of the required functionalities stated before, it cannot be used for context retrieval due to several weaknesses. For instance, when extracting context from multiple tables, queries become complex since a number of joins might be necessary.

Moreover, pure SQL does not support semantic annotations and does not provide a common understanding of the context information. Furthermore, the programming API of CML does not address the retrieval of context information with heterogeneous representations. Lastly, this approach does not fully support complex reasoning and pre-processing functions.

Another SQL-based query language that uses a relational database is presented by Feng [41]. They designed a query language for an ambient intelligent environment, which utilises contextual data to identify data retrieval conditions in a relational database. Since this approach is based on the relational database, it suffers from similar drawbacks identified for CML. In general, works that use native SQL for context retrieval are not suitable for context data management since they are limited to relational databases.

Schreiber et al. [42] proposed a framework to configure and manage pervasive systems, called PerLa. PerLa also adopts the database metaphor and uses an SQL-like query language for context retrieval. PerLa queries support both data acquisition and context retrieval by providing three types of queries: Low Level Queries (LLQ), which describe the behaviour of nodes, and determine the data selection criteria, the sampling frequency and the computation to be performed on sampled data; High Level Queries (HLQ), which determine the high-level elaboration involving data streams coming from multiple nodes, and Actuation Queries (AQ), which can modify devises’ parameters. Similar to Contory, the main shortcoming of PerLa is lack of support for expressing and distinguish the entity of interest in a query. Furthermore, PerLa does not support domain-based standards and has a very limited support for processing context data.

The most recent work in the area of SQL-based CQL is presented by Chen et al. [43], who proposed a new SQL-based CQL that supports both pull-based and push-based queries. This work introduces some useful ideas and concepts. Their work supports continuous queries with compound conditions for accessing contextual information from various context entities. Furthermore, the authors claim that their work also supports contextual functions. However, they did not describe how the contextual functions can be represented.

**Scheme 1:**
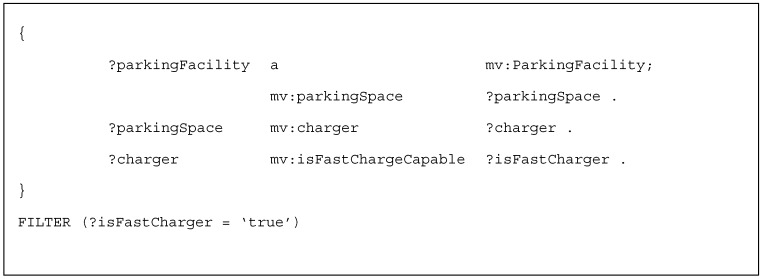
A basic condition expressed in SPARQL.

As it is demonstrated in [37], another powerful type of CQLs is RDF-based. The most well-established RDF query language is SPARQL. SPARQL has been used in many IoT platforms, such as OpenIoT, for querying contextual information. SPARQL [44] is a W3C standard proposal for an RDF query language whose syntax is inspired by SQL. It incorporates semantic concepts and ontologies into a SQL-inspired query language. SPARQL facilitates querying concepts of an entity, but it is not intended to be used for querying complex data constructs with several levels of nesting [45], which is commonly used in context-aware IoT applications. To clarify, consider the basic SPARQL condition presented in Scheme 1. This example presents an equality expression, which can be used to find all the parking facilities that have a parking space with fast charging points. In this example, the parking facility is defined based on mobivoc semantic vocabulary.

As it is shown in the scheme above, five lines of code with several variables are required to express this basic condition in SPARQL. As a result, queries easily become quite long and complicated which increases developers’ cognitive load. However, the same condition can be easily represented with only one line of code: parkingFacility.parkingSpace.charger.isFastCharger = ‘true’. Another drawback of SPARQL is its lack of support for defining custom aggregation functions. Furthermore, SPARQL does not provide a mechanism to define and query high-level context (i.e., situation). While it is possible to assume that context consumers can implement custom aggregation and situation reasoning functions as an additional layer of software, it contradicts with one of the main motivations behind developing a CMP which is providing a fast and easy way to query context and hide the complexity of low-level programming.

The MUSIC CQL proposed by Reichle et al. [45] is another well-known RDF-based CQL. Their work has a good support for querying contextual information. However, since MUSIC CQL can only represent context request from a single entity, it cannot express complex context queries (e.g., the query for the school safety scenario).

SOCAM (service-oriented context-aware middleware) framework [8] also provides an RDF-based CQL (based on OWL) for context retrieval. This language is capable of providing contextual data about IoT entities and the relationships between them by using an ontological approach. However, the main shortcoming of this work is its limited support for expressing complex queries.

The last category of context query languages that we review in this section is XML-based CQLs. A simple XML-based context description and query language was developed in the MobiLife project [46]. This CQL has a good support for string-based operators. In MobiLife, context queries are represented by identifying the value of a parameter, the timestamp of a parameter and different aspects of context such as accuracy and confidence. However, MobiLife does not have sufficient support for handling aggregation and reasoning functions. Furthermore, the dynamic discovery of context provider is not supported in this approach. In other words, context consumer has no information about the context providers before querying them.

Another work that uses XML-based language for querying context is the Nexus architecture [47,48]. Nexus is designed to facilitate the development of location-aware applications. The Nexus platform is designed on top of a common augmented world model that is described by AWML (Augmented World Modeling Language) and can be queried using AWQL (Augmented World Querying Language). AWML is responsible for modelling the world as data objects. Furthermore, AWML can integrate metadata into the model to improve the discovery and selection of context services [48]. On the other hand, AWQL also provides a query language for extracting information from the AWML. Some of the strengths of AWQL queries are their support for generalisation and aggregation rules, nearest neighbour queries and spatial relationships [49]. However, this language does not provide sufficient flexibility to support complex queries and expression of different aspects of context.

Another significant context query language is NGSI language [50]. NGSI is the main interface of FIWARE project [51], which is one of the most advanced CMPs in terms of consistent development and market penetration. Furthermore, NGSI was recently used as the base for development of an ETSI NGSI-LD standard for context information management [52,53]. However, the NGSI language [50], suffers from a number of drawbacks. NGSI supports only one entity per query, which limits the expressivity, flexibility, and query performance, and it also adds network overhead. Moreover, NGSI has limited support for situation reasoning and monitoring. To address this, FIWARE has integrated the Esper Complex Event Processing (CEP) engine [54], which uses Esper EPL [55] to represent monitored situations. However, NGSI and Esper EPL are two disjoint technologies, and this increases the development and maintenance efforts. Such an approach also adds conceptual complexities as Esper EPL is a more generic technology and is not designed to support IoT context-aware environments.

### 3.5. Discussions

We present a comparative evaluation of 10 existing context query languages mentioned previously, based on the six main requirements for a context query language, which are identified in Section 2. Table 1 shows a comparision of the CQLs with respect to these requirements.

In our view, meeting all of these requirements is essential for a CQL. For example, as illustrated in Table 1, more than half of the existing CQLs (6 out of 11) only support context queries concerning a single entity. However, in real-life scenarios, the contextual information comes from different context sources (e.g., smart vehicles, mobile devices, sensors). Therefore, those CQLs that do not fully satisfy this requirement are not good candidates for our objective.

Furthermore, another important aspect which needs to be appropriately addressed in designing a CQL is the support of interoperability. More precisely, without a common understanding (i.e., context model), smart entities (context providers and consumers) cannot communicate and exchange context with each other. Therefore, it is vital for a CQL to provide a context model that can be converted into different data models as required (as it should support existing context-aware systems). As depicted in Table 1, only ContextML [56] supports both requirements 1 and 2. However, this CQLs fail to meet requirements 4 and 5.

It can be seen that none of the existing CQLs fulfils all the requirements, whereas most of the approaches failed to meet the first two requirements. Moreover, to the best of our knowledge, none of these languages is known outside the research community and is not used in real environments. Furthermore, none of these languages has become a widely adopted standard, while such a standard is fundamental nowadays [52].

## 4. Context-as-a-Service (CoaaS)

In this section, we will first explain the big picture of CoaaS platform and provides the preliminary definitions, which are required the following discussions. Furthermore, a brief overview of CoaaS reference architecture is provided in Section 4.2.

### 4.1. CoaaS Vision and Definition

**Figure 2:**
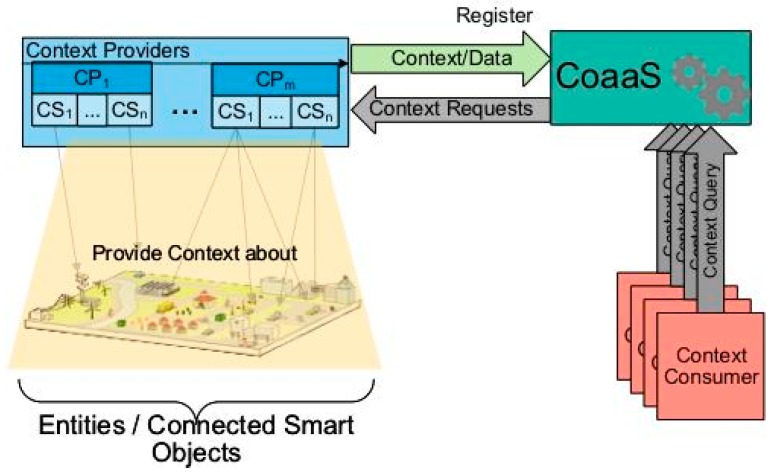
CoaaS big picture.

In this subsection, we will discuss the vision of CoaaS and define the basic definitions that are used in this paper. CoaaS is a context management platform, which has been developed as a step towards operationalising context-awareness in the IoT domain. The main motivation behind developing CoaaS is to facilitates context exchange between IoT entities, namely context providers and context consumers. In our terminology, any device or system that can provide context (or any relevant data that can be used to infer context) is referred to as a context provider (CP). Similarly, we define context consumers (CC) as any devices or systems that require contextual information. CoaaS can retrieve data about IoT entities by sending requests to corresponding providers. It can also process streams of context updates, which context providers are sending to the platform. Context updates contain updates of the entities’ states and are processed by CoaaS to monitor situations. The big picture of the CoaaS platform is shown in Figure 2.

As mentioned earlier, context is the information that can be used to characterise the situation of an entity [1]. Entities can be persons, locations, or objects which are considered to be relevant for the behaviour of an application. An entity can be characterised by a set of parameters, known as context attributes.

Definition 1—Entity and Context Attribute: In context-aware systems, an entity (denoted by $E_{k}$) accounts for a physical or virtual object (such as a person, car, electronic device) that can be associated with one to many context attributes (denoted by ${\mathrm{ca}}_{i}$) which can be any type of data that characterises this entity.

For example, a ‘car’ entity can have a location, speed, fuel level, number of available seats, model, and manufacturer as its context attributes.

Definition 2—Context Service: A context service (denoted by ${\mathrm{cs}}_{j}$) provides contextual information about a particular entity. Context service can be represented as a triple: 〈E, CAs, Ps〉 where E denotes the related entity, CAs is a set of provided context attributes, and predicates (denoted by Ps) form a set of logical expressions defined over CAs.

For example, a smart garage (which is a context provider) can provide a context service to deliver context attributes such as cost, available facilities, and time limit (contextual information) about available carparks (entity) in a specific location. Furthermore, the working hours of this garage are from 8 a.m. to 8 p.m. during weekdays, and 10 a.m. to 10 p.m. during weekends (complex context attribute). This context service description is represented as

$$cs_{1}:\;\langle E_{1},\;CAs_{1},\;Ps_{1}\rangle$$

where

$$\left\{ \begin{array}{c} E_{1}:carpark\\ CAs_{1}:\left\{\cos t,\;\mathrm{available}\;\mathrm{facilities},\;\mathrm{time}\;\mathrm{limit}\right\}\\ Ps_{1}:\\ location=LocA\;\wedge \\ (\left(wokingHours\;between\;8:00\;and\;20:00\;\wedge weekdays\right)\vee \\ \left(wokingHours\;between\;10:00\;and\;22:00\;\wedge weekdays)\right) \end{array}\right.$$

A context consumer is interested to collect contextual information about a particular entity with specific characteristics. To achieve this goal, the context consumer will issue a Context Query.

Definition 3—Context Query: Context query is a request for contextual information (either context attributes or high-level context inferred from context attributes) extracted from one or many entities.

For example, a smart vehicle can issue a context query to retrieve the cost, location, and number of available spaces (contextual information) of the best available parking facilities (entity of interest) near the driver’s meeting location based on his/her preferences. This query contains three main entities, namely parking facility, smart vehicle, and driver.

Each context query can be split into several sub-requests where the final result of the query will be computed based on the contextual information retrieved from results of these sub-requests by aggregating their results, providing them directly without changes, or using them to infer a higher-level context.

Definition 4—Context Request: A context request (denoted by $cr_{i}$) represents a request for contextual information about a particular entity. Context request can be represented as a triple: 〈E, CAs, Ps〉 where E denotes the entity of interest, CAs is a set of requested context attributes, and Ps is a set of predicates, which are defined over CAs using logical expressions.

The aforementioned context query for finding carparks can be broken down into three context request, one for each entity. The first request will be issued to retrieve context about the driver, the second request will be issued to identify the smart vehicle, and the last context request will be issued to fetch information about available parkings. These context requests are represented as

$$cr_{1}:\;\langle person,\;\left\{meeting,\;parking\;preferences\right\},\;\left\{driver\;id=101\right\}\rangle \;cr_{2}:\;\langle car,\;\left\{location,\;width,\;height,\;length\right\},\;\left\{VIN=202\right\}\rangle \;cr_{3}:\;\langle parking\;facility,\;\left\{location,\;cost,\#avilable\;spots\right\},\;\{distance\;\left(meeting.location,parking.location\right)<500\}\rangle$$

### 4.2. CoaaS Reference Architecture

While a detailed description of the CoaaS architecture and foundations lay beyond the scope of the current paper, its fundamental concepts are summarized in the current sub-section.

**Figure 3:**
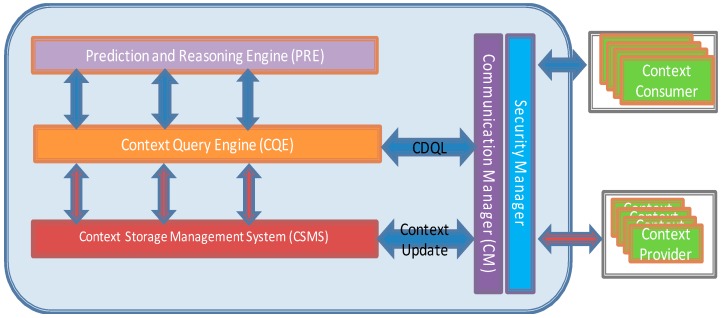
CoaaS reference architecture.

Figure 3 shows the reference architecture of the CoaaS framework, which is composed of four main components, namely (i) Security and Communication Manager, (ii) Context Storage Management System (CSMS), (iii) Context Reasoning Engine (CRE), and (iv) Context Query Engine (CQE). In the rest of this section, a brief description of each of these main enabling components is presented.

The ‘Communication Manager’ is responsible for initial handling of all the incoming and outgoing messages, namely context queries, context updates, and context responses. This module acts as a proxy and distributes all the incoming messages from CPs and CCs to corresponding components. To guarantee the privacy and security of CoaaS, this component is linked to the ‘Security Manager’. The ‘Security Manager’ module firstly checks the validity of incoming messages and authenticates requests. Moreover, the Security Manager checks whether the context consumer has access to the requested context service or not (authorization). Lastly, it is also responsible for monitoring all the incoming messages to identify any suspicious patterns, such as distributed denial- of-service (DDoS) attacks.

‘CSMS’, which is described in detail in [57], has three main objectives. First of all, it stores descriptions of context services and facilitate service discovery. Secondly, it caches contextual information to ensure reasonable query response time and deal with problems like network latencies and potential unavailability of context sources. Thirdly, the process of deriving context is based on knowing patterns and history as well as predicting future context to enable proactive adaptation.

The main task of the ‘Context Reasoning Engine’ is to infer situations from raw sensory data or existing primitive low-level context. It is a common need in many context-aware IoT applications to query about the situation of a context entity or trigger a query when a specific situation is detected. Situations can be seen as high-level context that is inferred from multiple low-level contexts [58].

**Figure 4:**
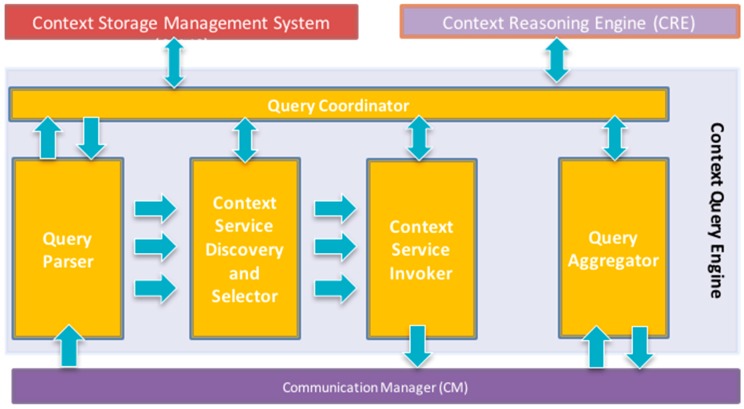
Context query engine architecture.

‘Context Query Engine (CQE)’ is mainly responsible for parsing the incoming queries, generating and orchestrating the query execution plan, and producing the final query result. Furthermore, this component also takes care of fetching required data from context providers on demand. As it is shown in Figure 4, CQE has five main components, namely Query Parser, Query Coordinator, Context Service Discovery and Selector (CSDS), Context Service Invoker, and Query Aggregator.

When a query is issued to CoaaS, after passing the security checks, it will be sent to the Query Parser. The Query parser has three main responsibilities: (i) parsing the incoming queries, (ii) break them into several context requests, and (iii) determine the query’s execution plan. Then, the parsed query plus the execution plan will be passed to Query Coordinator. The Query Coordinator plays an orchestrator role in CQE. This module is responsible for managing and monitoring the whole execution procedure of a context query.

In the next step, context requests will be pushed into the CSDS. This module is in charge of finding the most appropriate context service for an incoming request. This component consists of two parts: context service discovery and service selector. Context Service Discovery, which is implemented as a part of CSMS, finds context services that match the requirements of a context request. It will pass the descriptions of the candidate services to the Service Selector. Then, Service Selector returns a sorted set of the best available context services that can satisfy requirements of a request considering different metrics such as Cost of Service, and Quality of Service.

After selecting the best eligible context provider for each context request, requests will be passed to the Context Service Invoker. This component is responsible for fetching context from the corresponding context provider to retrieve the required contextual information and pass the retrieved information the query aggregator. Finally, the Query Aggregator combines the results of all the context requests and forms the final result of the query. Furthermore, the retrieved context might be used by the CRE to produce high-level context.

## 5. Context Service Description Language

In this section, we describe our proposed Context Service Description Language (CSDL) [59]. CSDL is a JSON-LD-based language that enables developers of context services to describe their services in terms of semantic signature and contextual behavioural specification; where the semantic signature defines the service name, number and types of its parameters, and the type of its output, and the contextual behavioural presents the context of entities provided by the service.

Furthermore, CSDL allows developers to describe their services using a standard language. CSDL enables the fast development of IoT applications that can discover and consume context services owned and operated by different individuals and organisations. For describing the semantics of context services, we adopted Web Ontology Language for Services (OWL-S) [31] which is a W3C recommendation, as the basis of CSDL. OWL-S is an ontology language, which is developed based on the Web Ontology Language (OWL) to enable automatic discovery, invocation, and composition of web services. However, as OWL-S was initially designed for describing web services and does not support the semantic description of context, we extended the OWL-S by adding the context description of the entities associated with context services.

**Figure 5:**
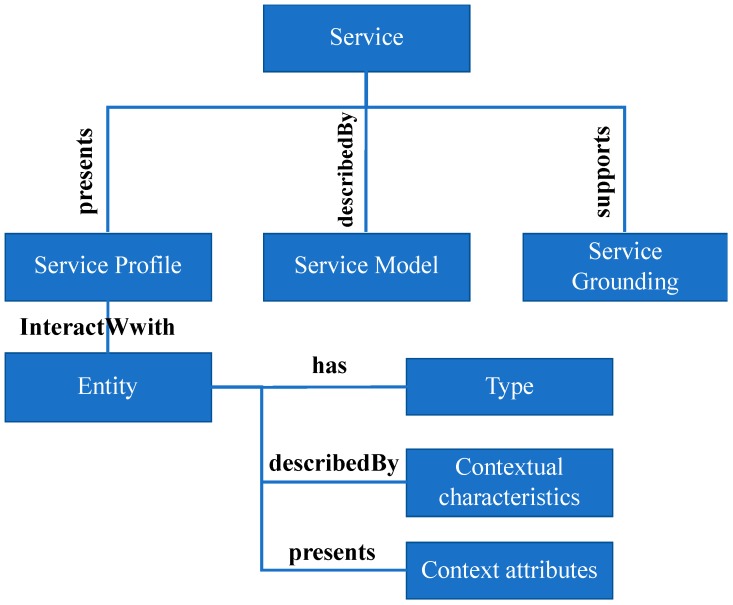
Structure of CSDL.

**Figure 6:**
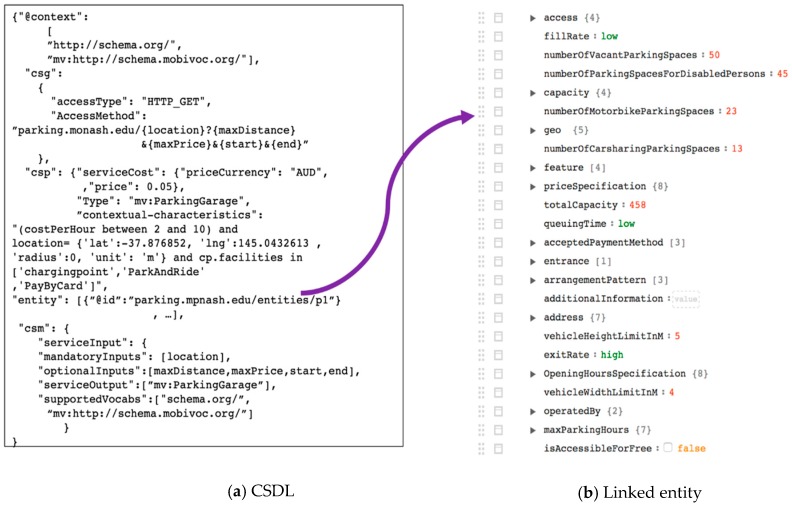
An example of service description in CSDL.

As shown in Figure 5, CSDL consists of three main components: (i) Service Profile, (ii) Service Grounding, and (iii) Service Model. Service Model gives a detailed description of a service signature, namely its input and output, and identifies the semantic vocabularies that are supported by the given service. Service Grounding provides details on how to interact with a service. This component identifies which type of communication needs to be used to call the service (e.g., HTTP get, XMPP, Google Cloud Messaging). Furthermore, based on the type of the communication, it will provide other required information to make the service invocation possible (e.g., URI in the case of HTTP get). Lastly, Service Profile is used to make service advertising and discovery possible. This component indicates the type of the entity that a service interacts with. Furthermore, it defines the context-aware behaviour of the service. Figure 6 shows an example of a service description in CSDL. This context service provides information about parking facilities located in Monash University.

## 6. Context Definition and Query Language (CDQL)

We describe our proposed Context Definition and Query Language (CDQL) in this section. CDQL consists of two main parts, Context Definition Language (CDL), and Context Query Language (CQL). The query part (CQL) provides the means for flexible and straightforward access to the available data. The definition part of the language (CDL) describes high-level context and situations.

### 6.1. CQL

**Figure 7:**
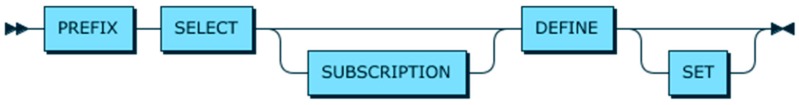
CQL production rule visualisation.

To fulfil all the discussed requirements for querying and sharing context (as discussed in Section 2 and Section 3) between entities in the IoT environment, we propose a novel query language called CQL. Figure 7 presents the production rule and highlights the core elements of this language. As the figure shows, CQL has three mandatory clauses, which are *PREFIX*, *SELECT*, and *DEFINE*; and two optional clauses, namely *SUBSCRIPTION* and *SET*. In the rest of this section, the details of each of these elements will be discussed. We will use an example to explain the syntax of CQL. The example under consideration expresses a query to find parking facilities with certain characteristics near a specific location.

**Scheme 2:**
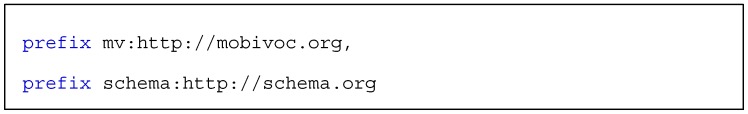
Example of PREFIX clause.

**Figure 8:**
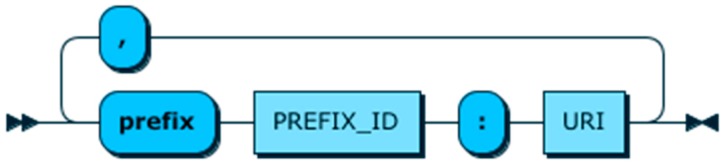
PREFIX clause production rule visualisation.

A CQL query starts with a prefix clause. The prefix clause is responsible for identifying the semantic vocabularies that are used in a query to facilitate interoperability (Requirement 2). Using semantic vocabularies and structures provides an easy and unambiguous way for a CQL developer to present their context queries. Furthermore, it helps CMPs to understand the information requested in a query and provide richer results. As it is illustrated in Figure 8, a prefix clause consists of two parts, a prefix id and an URI, which are separated by a colon. The prefix id assigns an identifier to a semantic vocabulary that will be used when it is needed to refer to it, and the URI refers to a semantic vocabulary. A CQL query can contain several semantic vocabularies separated by a comma. The Scheme 2 represents an example of *PREFIX* clause for the aforementioned parking query.

**Figure 9:**
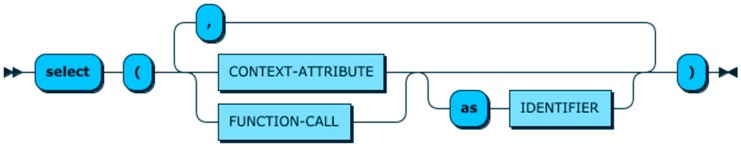
SELECT clause production rule.

The second mandatory clause of CQL is *Select*. This clause determines the query response structure. As shown in Figure 9, each context query can return a set of values as the query result, where each value can be represented as either a *CONTEXT-ATTRIBUTE* or a *FUNCTION-CALL*.

*A CONTEXT-ATTRIBUTE* represents a feature of an entity. This element consists of two parts: *CONTEXT-ENTITY-ID* and *IDENTIFIER*. The *CONTEXT-ENTITY-ID* identifies the entity which the context attributes will be queried from. The value for this element can be any of the entities known to the IoT ecosystem. We provide a mechanism to define such entities through the *DEFINE* clause, which is explained later in this sub-section. The *IDENTIFIER* determines the type of context we are interested in, such as temperature, noise level, or any other type. Furthermore, it is possible to retrieve all the available attributes of an entity by using an asterisk (*) wildcard.

**Scheme 3:**
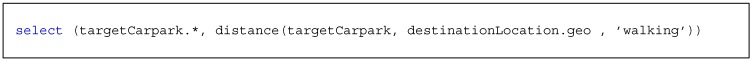
Example of SELECT clause.

The second possible element in the *SELECT* clause is a *FUNCTION-CALL*. This element allows querying high-level context, which is one of the requirements (Requirement 4) of a context query language. In CQL, reasoning and aggregation techniques are encapsulated as functions, referred to as *CONTEXT-FUNCTION*. A detailed explanation of *CONTEXT-FUNCTIONs* is provided in the next sub-section. *CONTEXT-FUNCTIONs* can be easily integrated into a query using the *FUNCTION-CALL* statement. The *FUNCTION-CALL* has four components: *PACKAGE-TITLE*, *FUNCTION-NAME*, *ARGUMENTs*, and *IDENTIFIER*. A *PACKAGE-TITLE* is an optional element that will be only used when the user wants to access a function defined inside a package. In this case, it is required to identify the namespace that the function belongs to. On the other hand, a *FUNCTION-NAME* is a mandatory module and determines the context function that needs to be applied to a set of arguments. The function’s argument can be a *CONTEXT-ATTRIBUTE*, a *CONTEXT-ENTITY*, or a *FUNCTION-CALL*. Scheme 3 represents an example of a *PREFIX* clause for the parking query. The first argument in this example is targetCarpark.*, which represents all the available attributes of an entity with ‘*id*’ equals to targetCarpark. The second argument is a *FUNCTION-CALL* that is used to calculate the walking distance between the selected carparks and the driver’s destination.

**Figure 10:**
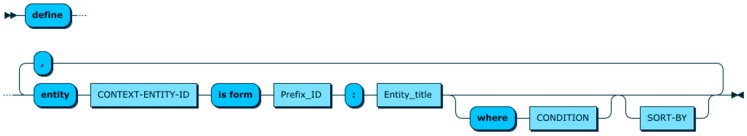
DEFINE clause production rule.

The last mandatory element of CQL is the *DEFINE* clause, which is represented in Figure 10. This clause allows querying contextual information from multiple entities (Requirement 1) by identifying the entities (one or several) that are involved in a query. In CQL, each entity is represented using four elements, *CONTEXT-ENTITY-ID*, ENTITY-TYPE, *CONDITION*, and *SORT-BY*.

The *CONTEXT-ENTITY-ID* assigns a name to an entity, which will be used when referring to the entity (e.g., in the *SELECT* clause).

The *ENTITY-TYPE* defines the type of an entity (e.g., car, parking facility, or a smart home) and consist of two parts, the *PREFIX-ID* that refers to a semantic vocabulary defined in *PREFIX* section, and a title, which represents the exact entity.

**Figure 11:**
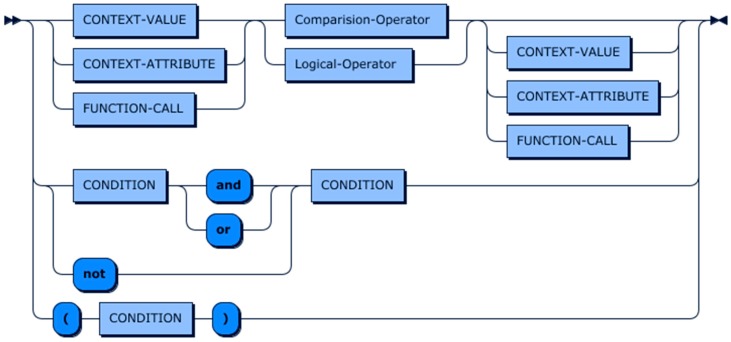
Condition clause production rule.

The *CONDITION* clause provides a guideline on how to filter out unwanted context entities from a large number of available entities. The *CONDITION* allows representing compound predicates that consist of several constraints connected by logical operators (AND/OR). These constraints define characteristics of the entity of interest. A constraint can be applied either to low-level context (*CONTEXT-ATTRIBUTE*), high-level context (*FUNCTION-CALL*), meta-data about context (e.g., freshness), or a simple value represented as a string or number. Furthermore, it is possible to combine multiple conditions into a compound condition by using the AND and OR operators. Figure 11 shows the production rule of the *CONDITION* clause. Please note self-referencing is used in this figure to represent compound conditions.

**Figure 12:**
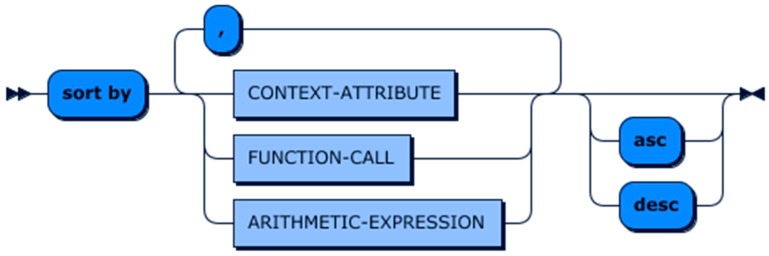
SORT-BY clause production rule.

Lastly, the *SORT-BY* clause is used to sort the retrieved entities in ascending or descending order. The syntax of this clause is presented in Figure 12. As this figure shows, this clause allows users to sort the result of each context request based on one or more values, where values can be either a *CONTEXT-ATTRIBUTE*, a *FUNCTION-CALL*, or an *ARITHMATIC-EXPRESSION*.

**Scheme 4:**
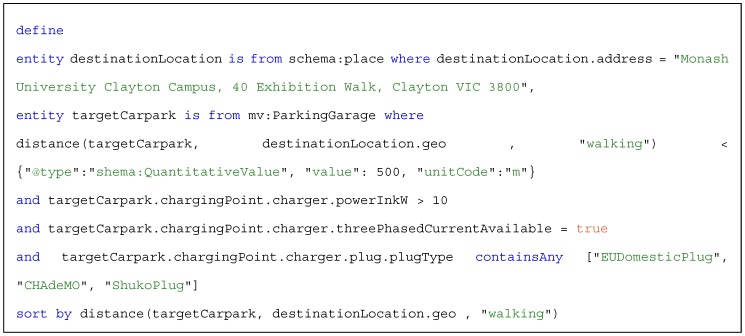
Example of DEFINE clause.

An example of *DEFINE* clause based on the parking query is shown in Scheme 4. This example consists of two entities, “destinationLocation” that identifies the destination’s location of the driver and “targetCarpark” that represents parking facilities with specific characteristics based on user preferences. As this example shows, attributes of one entity can be used in the definition of another entity.

**Scheme 5:**
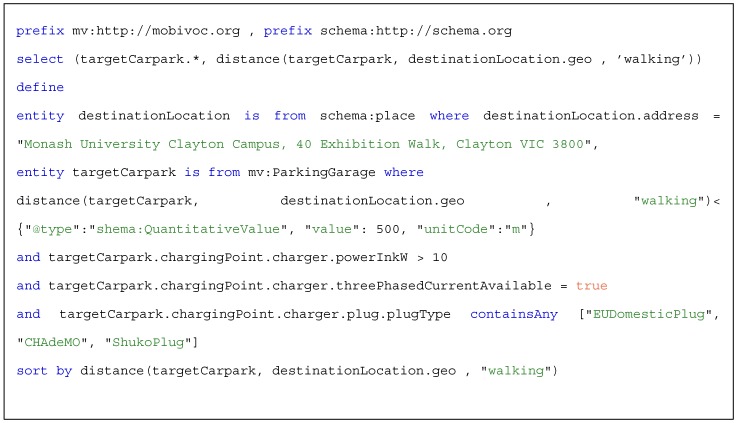
Example of a pull-based query.

So far, we introduced all the mandatory clauses of CQL. Using these clauses, a context consumer can issue complex context queries concerning various contexts entities and constraints, which will be executed only once immediately after the query has been issued. We refer to this type of queries as pull-based queries. Scheme 5 presents the full example of a pull-based query, which will be issued to retrieve all the available parking with specific characteristics close to a specific location.

As mentioned earlier, a common requirement in many context-aware IoT applications is to monitor IoT entities, discover situations’ changes, and adjust to them automatically. Therefore, we introduced the *SUBSCRIPTION* clause to address this requirement (Requirement 5). The *SUBSCRIPTION* clause supports the representation of periodic (e.g., check the temperature of a room every 10 min) and event/situation-based (e.g., when the temperature is more than 10 °C) context queries. Using this clause, a context consumer can receive periodic updates about the real-time state of an entity or subscribe to a specific situation. The result of the query will be sent back to the consumer asynchronously when the defined situation is detected. We refer to such queries as PUSH-based queries. In CDQL, to represent situations, we designed a specific syntax that supports rule-based reasoning, uncertainty handling, temporal relations, and windowing functionality. The syntax will be explained in the next sub-section.

**Figure 13:**
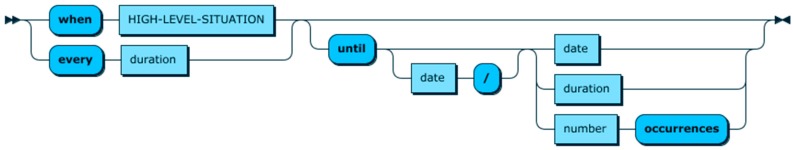
SUBSCRIPTION clause production rule.

The syntax of the *SUBSCRIPTION* clause is depicted in Figure 13. As this figure shows, the *SUBSCRIPTION* clause consists of either a *WHEN* or EVERY statement. Furthermore, it has an optional statement that is called *UNTIL*.

**Scheme 6:**
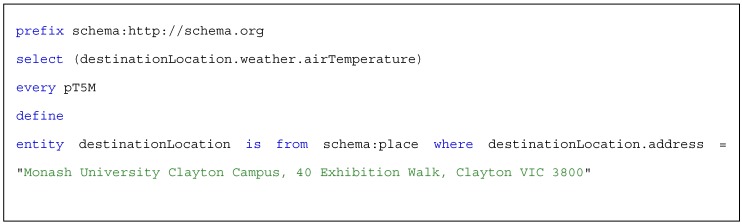
Example of a basic push-based.

The *EVERY* statement is designed to represent periodic queries by identifying the sampling interval for a context query. This statement starts with the ‘every’ keyword followed by a string which represents the sampling interval. To represents sampling intervals (i.e., duration) in CQL, we adopted ISO 8601 standard that provides a standard way to specify the amount of intervening time in a time interval in the format P[n]Y[n]M[n]DT[n]H[n]M[n]S[n]MS. In this format, [n] is replaced by the value for each of the date and time elements that follow the [n]. The capital letters P, Y, M, W, D, T, H, M, S and MS are designators for each of the date and time elements. For example, “P1Y2M6DT8H7M15S20MS” represents a duration of “1 year, 2 months, 6 days, 8 h, 7 min, 15 s, and 20 milliseconds”. Date and time elements including their designator may be omitted if their value is zero. Lower order elements may also be omitted for reduced precision. An example of a basic push-based query with an *EVERY* statement is provided in Scheme 6. By issuing this query, the subscribed context consumer will receive updates (i.e., every five minutes) about the temperature of a specific location.

**Scheme 7:**
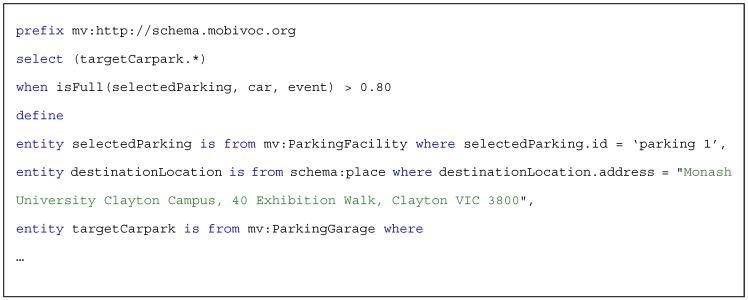
Example of using *WHEN* clause in a CDQL query.

The *WHEN* statement is the enabling element for situation-based queries. This statement starts with the ‘when’ keyword followed by a situation definition, which is expressed in a *HIGH-LEVEL-SITUATION* statement. Using this element, an IoT application can define and monitor their situations of interest. The *HIGH-LEVEL-SITUATION* statement is fully discussed in the next sub-section. The query represented in Scheme 7 is an example of a CQL query with a *WHEN* clause. This query expresses a request for monitoring specific parking spot that a car is driving to and suggests alternative carparks as soon as the situation “isFull” for the given carpark becomes true.

Lastly, the UNTIL statement indicates the timespan of the context retrieval by defining queries’ lifetime. As Figure 13 shows, the UNTIL statement provides three options to determine the query lifetime: the first option is to provide a DateTime struct to indicate the expiry date and time of a query, the second option is to provide the duration of subscription, and the last option is to provide the number of occurrences of query executions before it becomes deactivated. Furthermore, this statement can express the activation date and time of a subscription. In CQL, the DateTime struct is based on ISO 8061 standard and represented as “yyyy-mm-ddThh:mm:ss[.mmm]” (e.g., “2019-06-15T08:28:38”).

**Figure 14:**
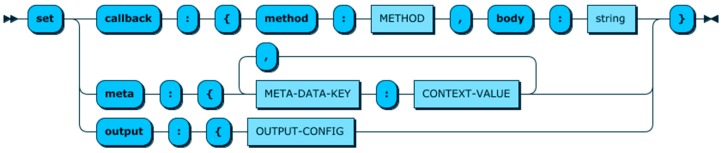
*SET* clause production rule.

The last clause of CQL is the *SET* clause, which is illustrated in Figure 14. This clause consists of three elements, namely CALLBACK, META, and OUTPUT.

The *CALLBACK* clause identifies how the result of queries should be sent back to the context consumers. This clause describes the callback method (e.g., HTTP Post) and other required fields (e.g., Callback URL and headers). Furthermore, this clause provides a mechanism to define the body of the message that will be sent back to the subscribed context consumer. As it is shown in Figure 14, the value for the ‘body’ attribute is a string, which can represent any custom messages in any format (e.g., JSON, XML, plain text, …). Moreover, it is possible to include any of the retrieved contextual information in the body string by using the ‘*$*’ prefix, i.e., “$*CONTEXT-ATTRIBUTE*”. If the ‘body’ attribute is not provided, all the entities and attributes defined in the select clause will be used as the message’s body. An example of using the *CALLBACK* clause is shown in Scheme 16.

**Scheme 8:**
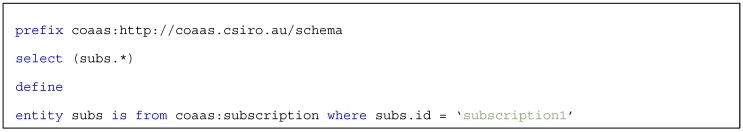
Example of querying the results of subscriptions.

The *CALLBACK* clause can be used for both push-based and pull-based queries. In the case of pull-based queries, it will allow context consumers to issue non-blocking queries and receive the result as soon as the execution of a query is finished. Regarding push-based queries, when the callback clause is presented, the result of the query will be pushed back into the subscribed entity as soon as the related situation is detected. When the callback is not provided, the result of the query will be temporarily stored, and the context consumer can pull the data by issuing a query similar to the one represented in Scheme 8, which indicates the subscription id.

**Scheme 9:**
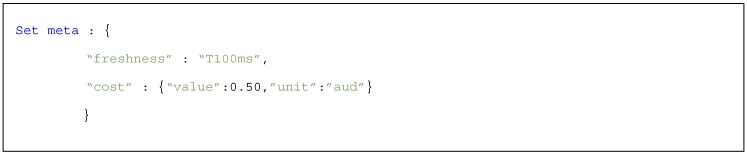
Example of META clause.

The META clause enables another essential requirement for a context query language, which is expressing different aspects of context, such as imperfectness, uncertainty, QoC, and CoC (Requirement 6). In other words, this clause allows user to set the minimum acceptable (or default) value for each metadata. For example, the query shown in Scheme 9 indicates that the minimum acceptable freshness for each context attributes is 100 ms and the total cost of query should be less than 50 cents.

**Figure 15:**
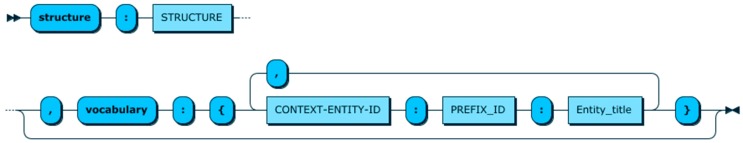
OUTPUT-CONFIG clause production rule.

Lastly, CQL allows developers of context query to define their preferred structure of output through the OUTPUT clause. The production rule of the OUTPUT clause is depicted in Figure 15. As it is shown in this figure, the output clause consists of two main elements, a *STRUCTURE* that identifies the output data structure (e.g., XML, JSON, or ODF), and a vocabulary that specifies which semantic vocabulary should be used for each context-entity.

In order to express the grammar of CQL, we used Extended Backus–Naur Form [60] (EBNF). The full grammar of CQL is represented in supplementary material.

### 6.2. CDL

As mentioned earlier, the reasoning and aggregation functionalities are supported in CQL through the notion of function. CDQL offers a rich set of built-in context-functions that can be easily integrated into context queries through a *FUNCTION-CALL*. Some of the most important CQL built-in functions are presented in supplementary material.

While built-in functions are sufficient for most common use cases, we believe it is mandatory for a CQL to support the definition of custom functions (Requirement 5), as these functions are usually application dependent and predefining a comprehensive list of them is not possible. As a result, we introduce the *CREATE-FUNCTION* clause in CDL to define aggregation and reasoning functions dynamically as part of the CDQL language.

**Figure 16:**
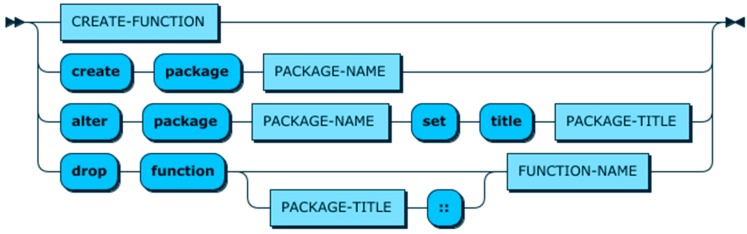
CDL production rule.

Figure 16 shows the CDL production rule. As depicted in this figure, CDL allows context query developers to create and remove *CONTEXT-FUNCTIONS*. Furthermore, it has three statement to create, alter, and drop packages. In general, packages in CDL are designed to organise functions and prevent function name collisions. Since the syntax of most statements in CDL are quite self-explanatory, except for *CREATE-FUNTION*. Hence, in the rest of this sub-section, we will focus on explaining the details of the *CREATE-FUNCTION* statement.

**Figure 17:**
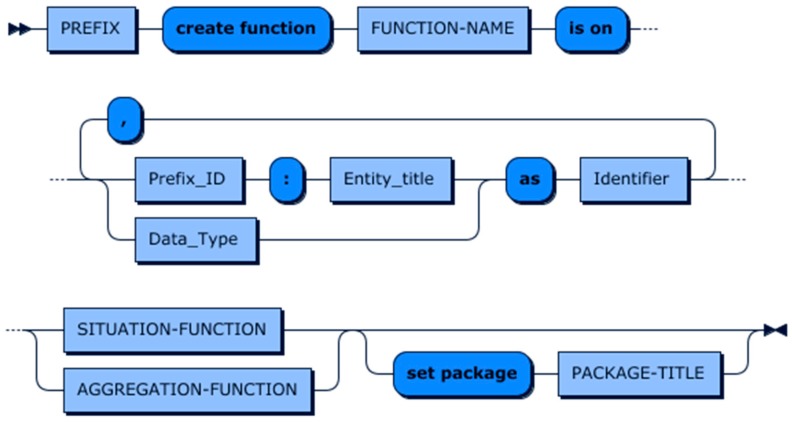
Create function production rule.

Figure 17 highlights the syntax of the *CREATE-FUNCTION* statement. As this figure shows, the *CREATE-FUNCTION* statement starts with a *PREFIX* clause, which identifies the semantic vocabularies used in the definition of the function’s parameters. It is followed by the ‘create function’ keyword and the *FUNCTION-NAME* construct that assigns a title to a context function and makes it accessible via this title.

The next keyword in the *CREATE-FUNCTION* statement is ‘is on’, which together with the *PARAMETER-DEFINITION* construct specifies the input parameters of a context function. This construct supports the definition of two types of parameters, which are *CONTEXT-ENTITY* and datatype. The supported datatypes in CDL are Number, Date, Time, DateTime, String, Array, and Object. Furthermore, the *PARAMETER-DEFINITION* construct assigns an id to each parameter using the ‘as’ keyword. In the *FUNCTION-CALL* statement, these parameters can be a *CONTEXT-ENTITY*, a *CONTEXT-ATTRIBUTE*, a *FUNCTION-CALL*, a literal value, or an expression, for example, it could be the arithmetic expression like ‘5*8’ or ‘parking.priceSpesification.price * meeting.duration’ where ’parking’ and ‘meeting’ are context entities.

After defining the signature of a function, the body of context function is constructed using either the *SITUATION-FUNCTION* construct or the *AGGREGATION-FUNCTION* construct. The details and syntax of these constructs is discussed in the rest of this section.

The last construct in the *CREATE-FUNCTION* statement is *SET-PACKAGE. SET-PACKAGE* is an optional construct and allows specifying the package to contain the function. If *SET-PACKAGE* is omitted, the context function will be placed into a default package, which has no name.

#### 6.2.1. Aggregation Function

As mentioned earlier, aggregation functions are usually application dependent, and it is not feasible to define all possible functions for all domains in advance. As a result, CDQL supports definition of custom aggregation functions. In CDL, aggregation functions can be expressed in two different approaches.

**Figure 18:**
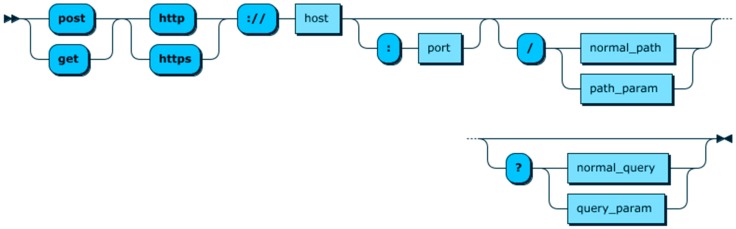
API-based aggregation functions.

The first approach is to provide aggregation functions through Restful API calls. This approach allows CDQL developers to register custom RESTful methods and use them in their context queries. The syntax of API-based aggregation functions construct can be divided into two sections. The first section of this construct expresses the endpoint of a Restful method by indicating the method type (i.e., get or post), the protocol (i.e., https or https), host address, and port number (if required). The second section, which consists of path parameters and query parameters, specifies the method of interest and its parameters. The production rule of the API-based function is presented in Figure 18. As this figure shows, functions can have several paths and query parameters, where each of them might be either a literal or one of the parameters defined in the *PARAMETER-DEFINITION* section. To distinguish parameters from literal, parameters are indicated by the dollar sign and curly braces (${car.speed}).

It is worth mentioning that if a method of an API-based aggregation function is set to ‘post’, all the parameters defined in the *PARAMETER-DEFINITION* section will be sent to the provided URI as a JSON object.

**Scheme 10:**
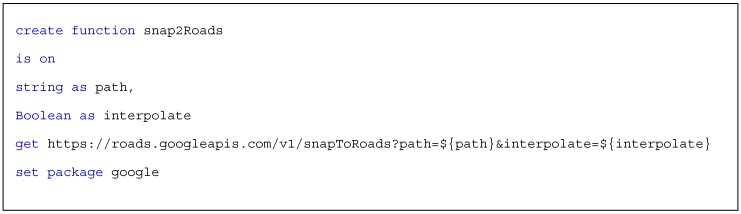
Example of CREATE-FUNCTION clause.

Scheme 10 shows a *CREATE-FUNCTION* statement that registers one of the Google maps’ APIs. This API takes up to 100 GPS points collected along a route and returns a similar set of data with the points snapped to the most likely roads the vehicle was travelling along.

**Scheme 11:**
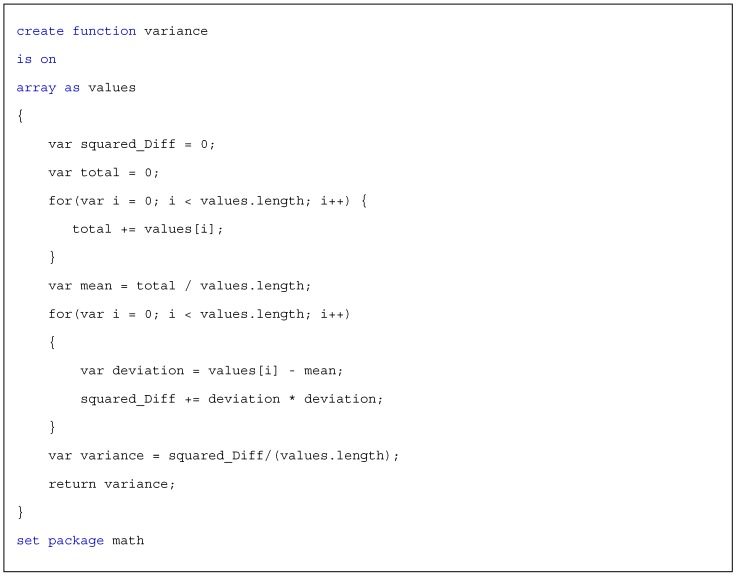
Example of creating a custom aggregation function using JavaScript.

The two main advantages of defining custom aggregation function as APIs are high reusability and ease of development. However, this approach might lead to a performance issue during query execution, especially when the volume of data that needs to be passed to the third-party APIs becomes large. Hence, to mitigate the performance issue in this type of use-cases, we introduced the second approach of defining custom aggregation functions. In this approach, CDQL developers can implement their custom aggregation functions using a scripting language, such as JavaScript or Python. This approach potentially has better performance compared to the first approach since the script will be executed locally (in the CMP) and there will be no communication overhead. Scheme 11 shows the implementation of the VARIANCE aggregation functions using the JavaScript language.

#### 6.2.2. Situation Function

In this section, we illustrate how *SITUATION-FUNCTIONs* are represented in CDL. First, we describe the situation model that serves as a basis for the definition of situations in CDL. Then, we explain the syntax of *SITUATION-FUNCTION* statement.

In CDQL, the situation representation and modelling are based on the Context Spaces Theory (CST) model [61] with some modifications and extensions to tailor our requirements.

The central notion in CST is the concept of situations. The CST model represents situations as geometrical objects in multidimensional space [61]. Such a geometrical object is called a ‘situation space’. A ‘situation space’ is a tuple of regions of attribute values related to a situation. Each region is a set of accepted values for an attribute based on a pre-defined predicate. For example, consider a situation labelled as ‘Good for Walking’ which indicates that the walking path from a suggested carpark location to driver’s destination is good for walking or not. This situation space can be characterised using several context attributes such as temperature, rain intensity, snow intensity, time of the day, the safety of the area, health status of a driver, age, etc. Furthermore, the acceptable regions of values for each context attribute should be defined, e.g., the lower and upper bounds of temperature.

In addition to basic concepts and techniques for situation modelling and reasoning, the CST model provides heuristics developed specifically for addressing context-awareness under uncertainty. These heuristics are integrated into reasoning techniques to compute the confidence level of the occurrence of a situation [62]. One of the main heuristics of the CST model is considering individual significance (weight) of each attribute. Weights are values from 0 to 1 assigned to every context attribute, and they represent the importance of each attribute in a situation, with a total sum of one per situation. In a simplified version of the example, only considering temperature, rain intensity, and safety of the area, the values 0.1, 0.3, and 0.6 can be assigned to these attributes respectively.

Moreover, CST assigns a *contribution* value to each region that indicates its level of participation in the occurrence of the situation. Back to our previous example, the regions and their confidence for the temperature attribute could include

$$Contribution_{\mathrm{temp}}=\;\{\begin{array}{c} 0.05\;Less\;than\left(-\right)5C\\ 0.6\;Between\;\left(-\right)5C\;and\;6C\\ 1\;Between\;6C\;and\;26C\\ 0.6\;Between\;26C\;and\;36C\\ 0.05\;More\;than\;36C \end{array}$$

Based on the discussion above, in CST, the confidence in the occurrence of a whole situation is defined as

$$\mathrm{Confidence}\;=\sum_{i=1}^{n}w_{i}*\;C_{i}$$

where $w_{i}$ represents the weight of a particular context attribute and $C_{i}$ stands for the contribution of the range to which the attribute’s i value belongs to.

Another way to represent situations is to combine several already inferred situations. However, the sequence of occurrence of such situations might play a role in situation inference. For example, a situation ‘S’ can be considered to be happening if the situation ‘A’ happened before the situation ‘B’, but not ‘B’ happened before ‘A’. This type of dependence is called ‘temporal relation’, and it is essential to include this feature in the situation description model. Since it is not directly supported in CST, we adopt Allen’s interval algebra [63,64] to enable the representation of such kind of relation.

Furthermore, a situation can be defined as a generalisation of similar events over a certain period of time. In simple words, situation A can be described as: “Situation A is happening if a particular sensor reading was in the range between X and Y during the last 30 min”. In this example, the “during the last 30 min” is an implicit usage of a common technique for data stream processing - a sliding window. A window can be defined as “a mechanism for adjusting flexible bounds on the unbounded stream in order to fetch a finite, yet ever-changing set of tuple” [65].

Similar to the temporal relationships, the windowing functionality is not supported in CST. Therefore, in order to support this functionality, we integrated four types of windows into the situation description model, namely (i) sliding window, (ii) tumbling window, (iii) hopping window, and (iv) eviction window. Until now, we covered the core concepts that form the foundation of situation description in CDL. In the rest of this section, we will present the syntax of Situation Description Statement (SDS). SDS provides two statements for depicting situations, namely the *CST-SITUATION* statement and the *HIGH-LEVEL-SITUATION* statement.

**Figure 19:**
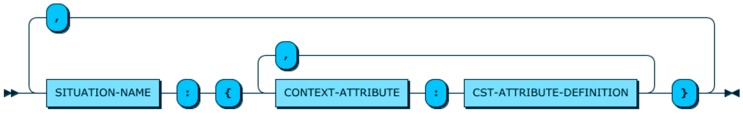
CST-SITUATION statement production rule.

The *CST-SITUATION* statement is purely based on Context Spaces Theory (CST) and describe situations in terms of their related context attributes combined with acceptable regions of values for each attribute. Figure 19 shows the syntax of CST-based situation description. As illustrated in the figure, a *CST-SITUATION* statement can have several situations, where each situation starts by assigning a name to it. In the next part, all the involved *CONTEXT-ATTRIBUTE*s and their corresponding values, which define the characteristics of the situation, should be listed.

**Figure 20:**
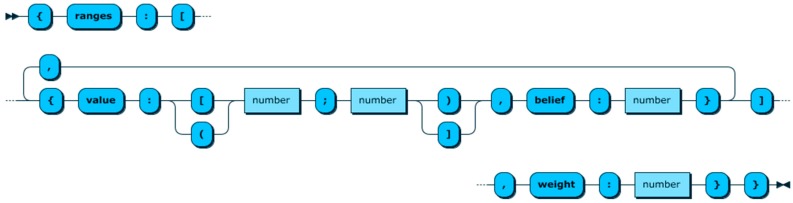
CST-ATTRIBUTE-DEFINITION.

The value of CONTEXT-ATTRIBUTEs is represented by the CST-ATTRIBUTE-DEFINITION construct that can be seen in Figure 20. This construct has two elements, ‘ranges’ and ‘weight’. The ‘Ranges’ defines the acceptable regions for an attribute by indicating the exact range, and the value of ‘belief’ that indicates the level of participation of an attribute in the occurrence of a situation, when its value is within the indicated range. The ‘weight’ construct identifies the importance of an attribute in a situation by providing a numeric value between 0 and 1.

**Scheme 12:**
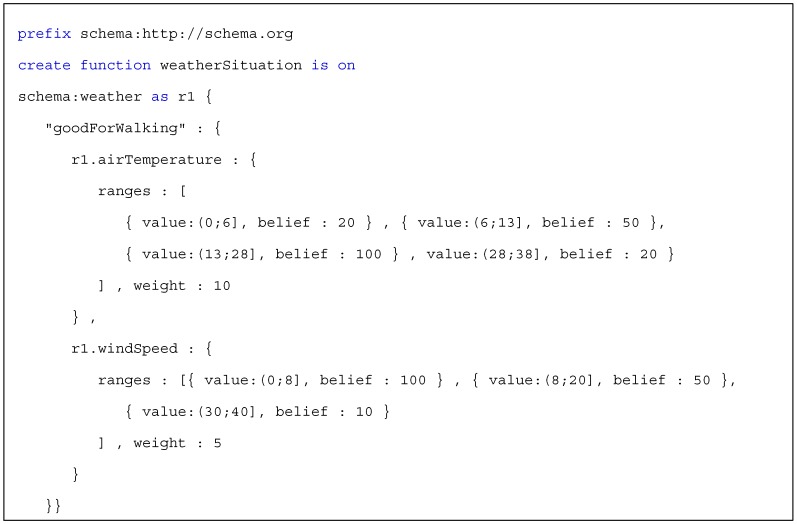
Example of example of CST-based situation function definition.

Scheme 12 shows an example of a situation function definition in CDL. This example expresses the aforementioned goodForWalking situation.

As mentioned earlier, the CST model does not support expressing situations that contain temporal relationships or windowing functions. Therefore, to express this kind of situations, we introduced the *HIGH-LEVEL-SITUATION* statement. This statement supports description of higher-level situations by describing the correlation of situations via temporal relationships and logical operators.

**Figure 21:**
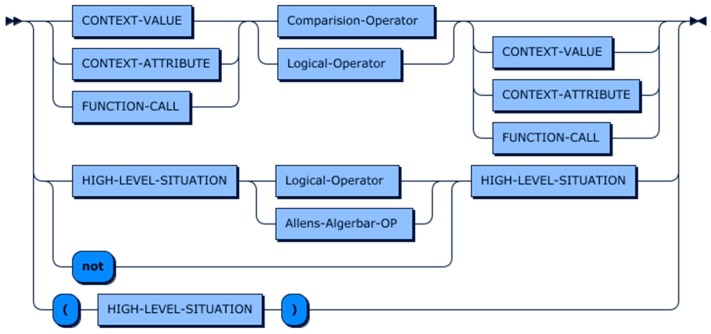
HIGH-LEVEL-SITUATION statement production rule.

**Figure 22:**
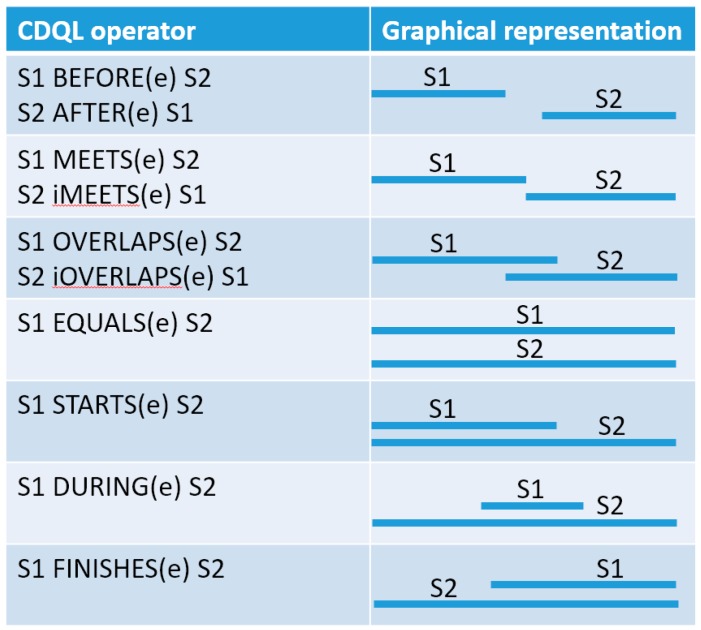
Allen’s algebra graphical representation (i stands for inverse).

The production rule of this statement is presented in Figure 21. As shown in the figure, the syntax of the *HIGH-LEVEL-SITUATION* statement is very similar to the *CONDITION* clause, with the only difference that the former allows connecting two high-level-situations with temporal relationships operators. In CDL, we adopted seven operators from Allen’s interval algebra, namely Before, Meets, Overlaps, Starts, During, Finishes, and Equals. The graphical representation of temporal relations between events is presented in Figure 22.

**Figure 23:**
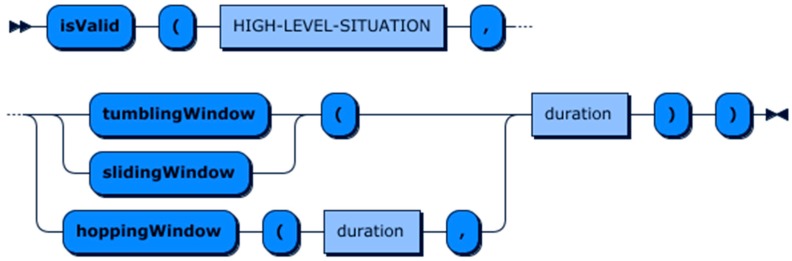
isValid function production rule.

Another concept that was mentioned earlier in this sub-section is windowing. To enable a query to express the validity of a situation over time, we introduced a new built-in function—‘isValid’. This function accepts a situation and a period of time as its inputs and returns the average confidence of occurrence of the given situation over a defined period. It enables both, the possibility to access historical trajectory of the situation, and, also, a sliding, hopping, tumbling, and eviction window functionality. The formal representation of using the ‘isValid’ operator is presented in Figure 23.

**Scheme 13:**
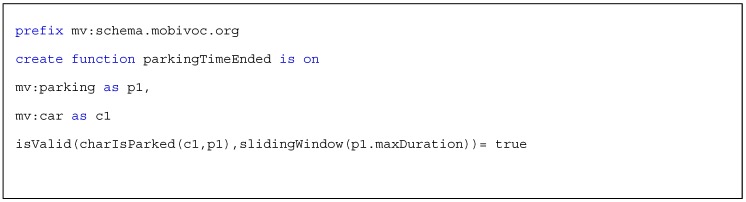
Example of isValid function.

An example of using the ’isValid’ operator for a real situation’s description is shown in Scheme 13 line 5. This SDS describes a situation when a period of parking exceeds the allowed maximum duration.

Furthermore, CDQL is enhanced with a rich set of statistical functions that can be used to improve the expressivity of the situation description -related part of the language. These functions accept a context attribute and a window as its input and return statistical information as the output. Some of the most essential CDQL’s statistical functions are provided in supplementary material.

## 7. Evaluation

In this section, we evaluate the proposed CDQL. First, we demonstrate the feasibility and applicability of CDQL by presenting exemplary queries for each of the use cases discussed in Section 2. Furthermore, for one of the use cases (i.e., smart parking recommender) we implemented a proof of concept application to show how CDQL queries can be utilised to develop context-aware IoT applications.

Secondly, to show the advantages of the proposed language compared to existing CQLs, we discussed how the aforementioned use cases can be implemented in the NGSI language, which is probably the most advanced existing CQL.

Lastly, to show it is possible to develop a CMP that uses CDQL language and is capable of dealing with the load in large-scale IoT environment, we conduct multiple experiments based on real-world and synthetic datasets. In all, the conducted experiments we used the CoaaS platform, which is the reference implementation of CDQL language.

### 7.1. Feasibility Demonstration

In this section, to exemplify the proposed context query language, we apply CDQL to the aforementioned use cases in Section 2, namely school safety, smart parking recommender, and vehicle preconditioning. We illustrate how CDQL can be used to represent and describe the context entities, their relationship, and context queries to fulfil the requirements we identified in Section 2. In this section, we mainly focus on showing how CDQL queries can be used to facilitate development of different use-cases. For the details on how CoaaS execute CDQL quires the reader can refer to our previous paper [13].

#### 7.1.1. Use Case 1: School Safety

**Scheme 14:**
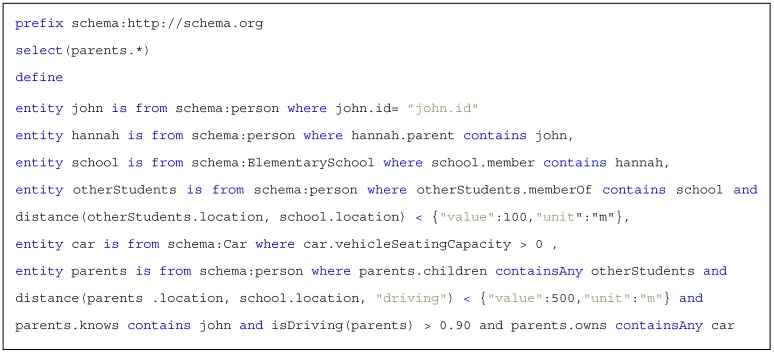
CDQL query for school safety use-case.

As we mentioned, CDQL can represent complex context queries concerning several entities. Furthermore, it also supports definition and querying of high-level context. To represent these functionalities, we will use the school safety use case, where John is late and looking for a trusted parent to pick up her daughter, Hanna. We start this query by defining the involved entities. As this query is designed to be executed by John’s device, we first define John by using his unique user ID. Now, by applying the Parenthood relationship, the entity that represents Hannah in this query can be defined. In the same manner, by using a membership relationship, we can use entity Hannah to define Hannah’s school. Now, as we define the entity that represents Hannah’s school, other school students can be defined as well. The two remaining entities for this query are car and parent. For representing cars, we need to add a constraint on the available number of seats. Then, as a final step, the parent entity can be defined by using the ownership relationship which indicates the selected persons who have a car with an empty seat for Hannah, and the parenthood relationship to show that the selected person is the parent of one of Hannah’s fellow students. Furthermore, another constraint should be added to check whether the selected person is close enough to school or not. Scheme 14 shows the complete CDQL query for this use-case.

This example clearly illustrates the power of CDQL to express very complex queries that need to acquire contextual information from several heterogeneous entities.

Two other important aspects for such a context query that access sensitive personal information are privacy and security. In Section 4, we briefly explained how the current implementation of CoaaS handles authentication and authorization. Hence, since these aspects are mostly handled by the underlying platform, not the language itself, they are not detailed here.

#### 7.1.2. Use Case 2: Smart Parking Recommender

The second use case that we study in this section focuses on the development of a smart parking recommender application that utilises context to suggest the best available parking. To implement such an application, several challenges need to be addressed. First of all, it is essential to have access to live data regarding the availability of different parking facilities. The fact that these facilities are owned by different providers (e.g., city administrators, building owners, and organisations) makes the process of data retrieval even more complicated. Furthermore, to be able to provide personalised suggestions to users, we need to consider additional factors, such as user preferences, car specifications, and weather condition. In addition, some of the data need to be inferred before being used. Addressing all these challenges needs a considerable amount of efforts, even for an expert team of software developers.

**Scheme 15:**
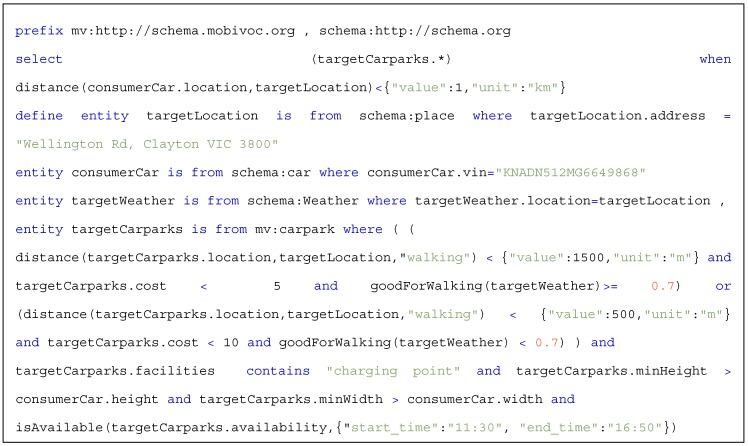
CDQL query for smart parking recommender use-case.

However, with the help of the CDQL language, all the above-mentioned context can be retrieved by issuing a CDQL query. To prove our claim, we developed an Android mobile application, which automatically provided suggestions about available parking spaces to drivers using real data. To achieve this goal, we composed a parameterised push-based CDQL query that will be triggered when the consumer’s car is getting close to the user’s destination. This query takes different contextual attributes such as weather conditions, walking distance, required parking facilities, and cost into account. Furthermore, this application provides an interface for users to enter their parking-related preferences in the application. Moreover, the application is connected via Bluetooth to an OBD II device which reads the sensory data (e.g., VIN, speed, and fuel level) coming from the car’s CAN bus. Then, the application takes this information, puts it in the query, and posts the CDQL query to CoaaS. Scheme 15 shows the query.

**Figure 24:**
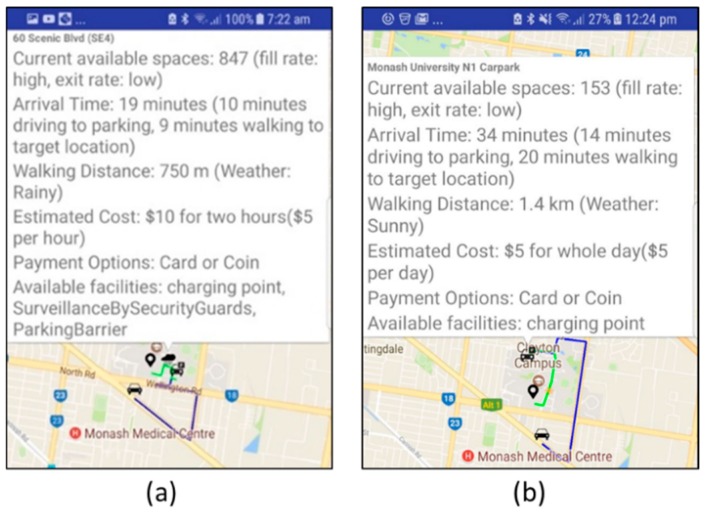
PoC parking application screenshot. (**a**) bad weather conditions; (**b**) good weather conditions.

Figure 24 shows the screenshots of the developed application in two different days with different weather conditions. In Figure 24a, the application suggested a more expensive parking with less walking distance because of the bad weather conditions. On the other hand, in Figure 24b, the application suggested a parking space that was cheaper but further away because it was a sunny day.

#### 7.1.3. Use Case 3: Vehicle Preconditioning

**Figure 25:**
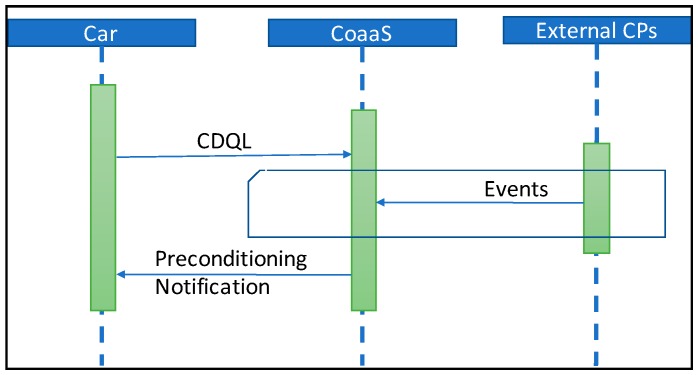
Pre-conditioning scenario workflow.

**Scheme 16:**
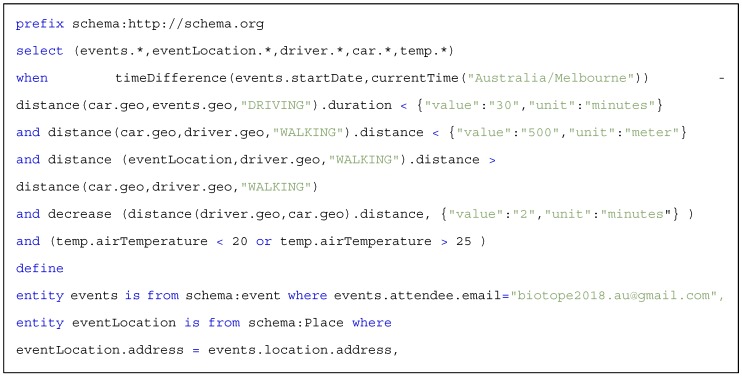
CDQL query for vehicle preconditioning use-case.

The last use case in this section is based on the vehicle preconditioning and shows how CDQL can be used to issue an actuation signal to turn on the car air conditioning system. This use case is conducted in a real environment using a BMW i3 car. Figure 25 shows the workflow of the experiment. The life cycle of this test is started by the car, which issues a PUSH-based CDQL query to CoaaS. This query, which is shown in Scheme 16, represents a complex situation that contains several entities such as the driver, car, parking location, and weather. Furthermore, the following conditions are expressed in the query to infer if the car is likely to be used in the near future or not:

- Is there an upcoming meeting where the driver is likely to use the vehicle?
- Is the driver in walking distance from the car? Is the driver walking towards the car?
- Is the distance between the driver and the car less than the distance between the driver and the meeting location?
- Is the distance between the driver and the meeting location out of walking distance?
- Is the temperature lower or higher than a certain threshold, so is the pre-conditioning necessary?
- Is the vehicle connected to a charging point? Is the battery level high enough for both pre-conditioning and driving to the next destination?

When CoaaS receives this query, it starts to monitor all the incoming events from external context providers that contain relevant contextual information about any of the entities mentioned above. Furthermore, it evaluates the occurrence of the situation defined in the query and notifies the car when the situation is detected.

To test the use case, we created a meeting event in the driver’s Google calendar, where the meeting location satisfied the mentioned criteria. We also enabled the smartphone application to send events containing the driver’s current location to CoaaS. CoaaS was able to detect these changes in real time and send the corresponding situation notification (actuation) to the context consumer (BMW backend server) to start the car’s climate control system as the driver started to walk towards the car.

In these demonstrations, we showed how complex use cases from different smart city applications can be implemented by issuing only one CDQL query. While implementing the whole use case from scratch requires a considerable amount of time and effort from the developers, using the proposed query language significantly ease the development of context-aware IoT applications. Developers only need to issue a CDQL query for querying and monitoring context of several IoT entities and detecting complex situations.

### 7.2. Comparison of CDQL with NGSI

As it is mentioned in Section 3, the most sophisticated existing context query language is NGSI. Therefore, to illustrate the advantages of the proposed context query language, we compare it with NGSI. To do so, we will first discuss how smart parking recommender use case and vehicle preconditioning use case can be implemented using NGSI language. Next, we will compare the implementation of these use cases in NGSI with CDQL and will discuss the outcome.

In the previous section, we showed how the required contextual information for parking recommender use case can be expressed with a single CDQL query. However, it is not possible to implement this use case with one NGSI query as NGSI is not expressive enough for such a complex scenario.

**Scheme 17:**
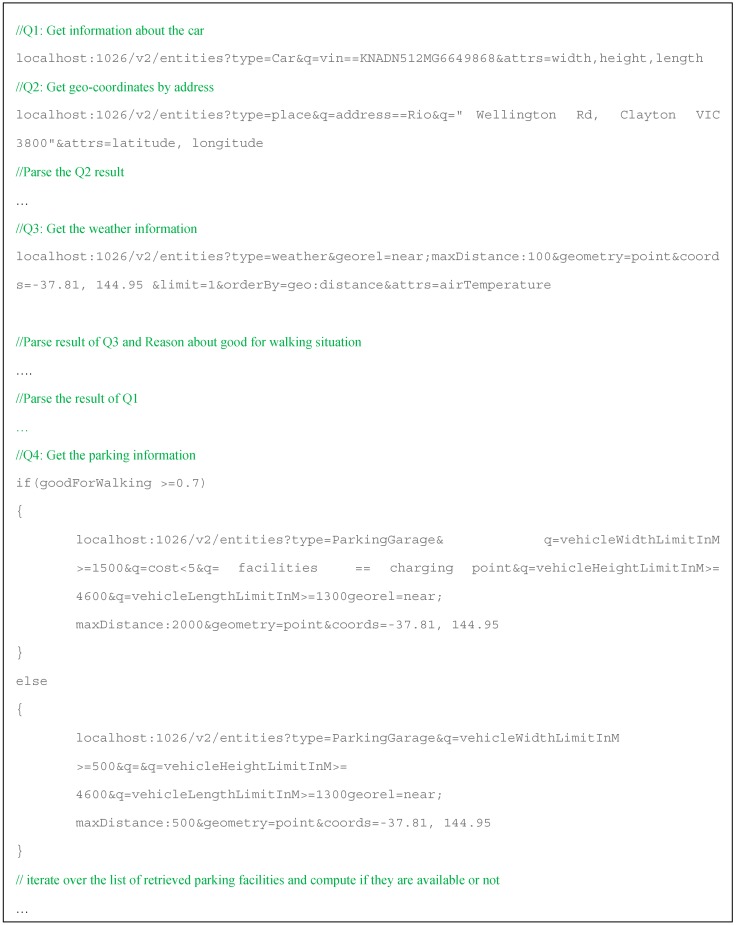
NGSI queries for smart parking recommender use-case.

Scheme 17 presents the pseudocode of implementing use case 2 using NGSI. As mentioned earlier, NGSI only supports querying one entity type per request. As a result, in order to implement this use case that involves several entities, four context queries are required to be implemented and issued.

Moreover, NGSI does not support context reasoning and custom aggregation functions. Therefore, it is not possible to integrate such functions (i.e., goodForWalking and isAvailable) in NGSI queries and it is responsibility of the developer of such an application to implement these functions. In addition, NGSI does not support ‘*OR*’ operator. Hence, if such functionality is needed, developers should implement several versions of a query and use ‘*if*’ statement to decide which one should be issued.

Another use case that we discussed in pervious section is vehicle preconditioning. Implementing this use case requires monitoring context of several entities (e.g., driver, car, and weather) and reason about if the precondition should be initiated or not. While NGSI allows monitoring changes in the context information, its subscription model is not sophisticated enough for this use case. First, NGSI subscription model only supports monitoring context of one entity type per subscription. Second, NGSI does not support situation inference and window functions.

**Scheme 18:**
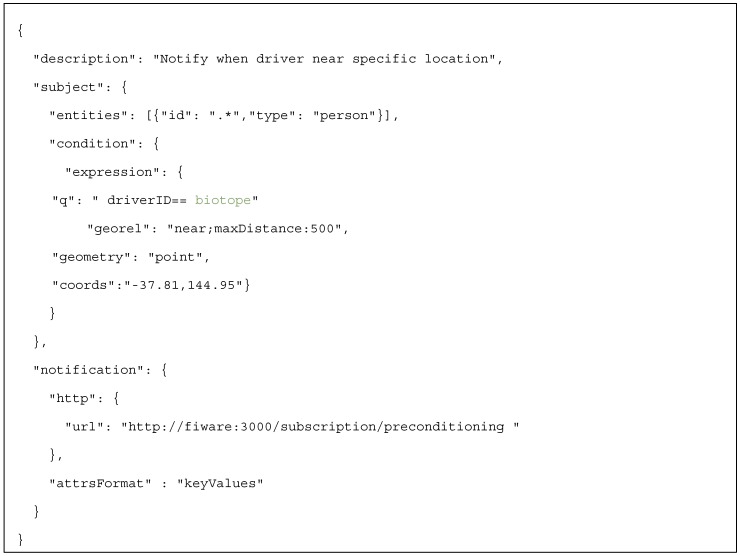
NGSI subscription.

Therefore, it is not possible to fully implement use case 3 using only NGSI without having this functionality at consumer’s side. Scheme 18 shows an example of NGSI query for subscribing to receive notification when the distance between driver and a specific location is less than 500 m.

As it is illustrated in these use cases, one of the main advantages of CDQL over NGSI is the support for expressing multiple entities in one query. As a result, several NGSI queries might be required to implement a use case that can be expressed with only one CDQL query. For example, in use case 2, the consumer performs four NGSI queries, causing extra time and network bandwidth for data transfer and processing. Moreover, having more quires makes the implementation and maintenance of context aware applications harder.

Furthermore, the lack of support of NGSI for querying more than one entity type in a request leads to increase in several other unavoidable drawbacks, namely (i) difficulty to avoid retrieving data which is intermediate and may not be really needed in the final result, (ii) difficulty to protect intermediate data from access, and (iii) difficulty to avoid network delays.

On the other hand, the CDQL not only address these shortcomings, but it has several other benefits compared to NGSI as well. For example, it is possible to integrate query optimisation to improve overall performance of the system.

Apart from the number of supported entities, CDQL provides several other functionalities that are essential for a CMP and not supported in NGSI, such as supporting aggregation functions, window functions, situation inference functions, and temporal relations, just to name a few.

Based on the discussion above, we can make a claim that CDQL can provide significant benefit for CMP platforms compared to NGSI.

### 7.3. Performance Evaluation

The main objective of this section is to illustrate that it is possible to develop a scalable CMP, which is capable of executing CDQL queries in near real-time. In order to achieve this goal, we implemented a prototype of CoaaS platform using Java EE technologies, which allows issuing CDQL queries through a RESTful interface. This prototype is running as a web application on Payara Server 5.182 where the maximum JVM heap size and maximum thread pool size are 16 GB and 500 threads respectively. The Payara Server is hosted on a virtual machine located in CSIRO Melbourne Cloud and running Debian GNU/Linux 8 (Jessie). The VM is running on an eight-core Intel(R) Xeon(R) CPU E5- 4640 0 @ 2.40 GHz instance with 64 GB RAM.

For our experiments, we used real parking data provided by the Melbourne city portal [66]. This dataset contains information from in-ground car parking bay sensors deployed in the Melbourne Central Business district. Update frequency of the dataset is two minutes, and the number of parking spaces is 2767. Moreover, since we wanted to test the scalability of CoaaS, we developed a script, which simulated more parking spots based on the aforementioned dataset.

We also developed a context provider simulator, which imitates the behaviour of IoT entities (i.e., driver location, car park status). Context updates are randomly generated in a way that each update has a 30% chance of triggering a subscription.

For all the experiments in this section, we used JMeter 4 to simulate and issue CDQL queries. We deployed the JMeter 4 in the same network where the CoaaS instance was running to minimise the network delay since we are only interested to measure the performance of the CoaaS.

#### Experiment 1

This experiment focuses on the performance evaluation of PULL-based queries. As results of the experiment is dependent on the infrastructure, especially on the application server, we conducted an initial test to find the maximum number of requests that an application server can handle. We found that in the current setup it is possible to serve a maximum of 570 HTTP POST requests per second.

At first, we studied the impact of query load to show how CoaaS performed when the number of concurrent queries increased. To this end, we conducted 13 tests by gradually increasing the query load (query per second) from 40 to 560, where the step size was 40. Each test was executed for 10 min and the average query response time was measured.

**Figure 26:**
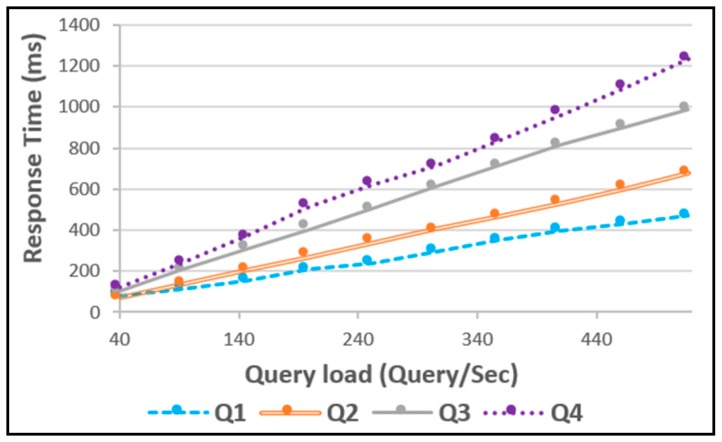
Query response time vs. input rate.

It is worth mentioning that during this experiment the number of registered parking spaces was equal to 2767. Moreover, to take the impact of the complexity of queries into account, we repeated this experiment using four queries with an increasing level of complexity. The first query (Q1) represented a search for a car park by providing its ID. The second query (Q2) was a location-based query, which searched for available parking spots near a specific coordinate. In the third query (Q3), we extended the previous query by taking the car specification (i.e., width, length, and height) into account, which required adding an entity representing the car in the query. In the last query (Q4), we added a situation reasoning function to the previous query. This function added the walking conditions between the destination and the car park into the scope. Results of the experiment are presented in Figure 26.

The result of this experiment shows the processing time of a query grows linearly with the increase in the query load. The increase in query complexity increases the steepness of the graph. However, it can be seen that even for the most complex query (Q4) while the incoming query load was 550 query/sec, the response time is close to one second. This response time is within the acceptable range for the most of IoT applications.

**Figure 27:**
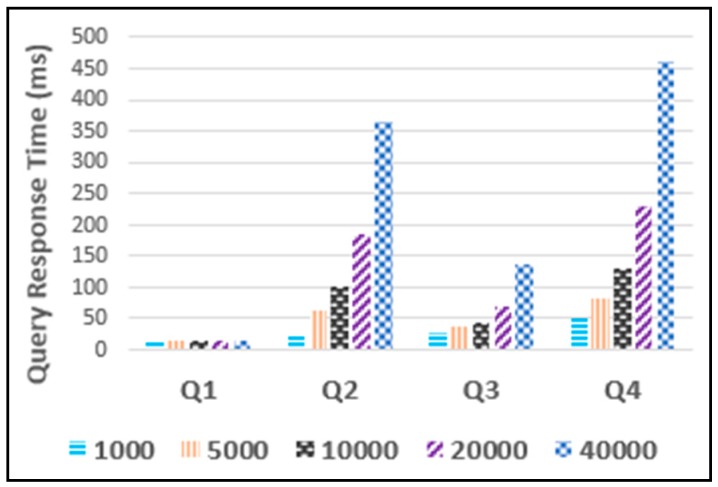
Query response time vs. number of registered entities.

Next, we designed another experiment to study the impact of the number of registered entities (i.e., parking spaces) on query execution time. In this experiment, we varied the number of registered parking spaces from 1000 to 40,000. The query load was equal to 100 query/s. Similar to the previous experiment, we ran this experiment four times according to the queries above. Furthermore, we ran each test for 10 min and measured the average query response time. Results of the experiment are presented in Figure 27.

As shown in the bar chart, the response time of Q1 remained unchanged while the query load increased. The reason is this query searches for a unique indexed attribute. However, in the case of other queries, we observed a linear growth of processing time. These queries are geolocation-based, and the number of entities can directly affect the search space. Interestingly, we observed Q3, which had more attributes than Q2, and had a lower response time. The reason for this effect is short-circuiting. Short-circuiting means the second argument of a logical expression is evaluated only if the first argument is not enough to determine the value of the expression.

#### Experiment 2

In this experiment, we focus on the evaluation of the push-based queries by conducting two sub-experiments to show how the CoaaS platform deals with the increase in the number of context updates and the number of subscriptions. In both experiments, we used the preconditioning push-based query, which was presented in Scheme 16.

The first experiment shows the impact of the number of incoming context updates on the execution time of push-based queries. To describe the results, we used the following metrics: *input rate*, *throughput*, *processing time*, *CPU usage*, and *memory consumption*.

The *input rate* denotes the number of incoming context updates per second.

$$Input\;Rate=\frac{Number\;of\;incoming\;Context\;Updates}{T_{end}-T_{start}}$$

The *throughput* depicts the number of context updates which were fully processed by the platform.

$$Throughput=\frac{Number\;of\;Processed\;Context\;Updates}{T_{end}-T_{start}}$$

*Processing time* shows the time, which is needed to process a context update from the time it reached the situation framework ($T_{in}$) until the moment it gets fully processed ($T_{out}$).

$$T_{\mathrm{Processing}}=T_{out}\;-T_{in}$$

**Figure 28:**
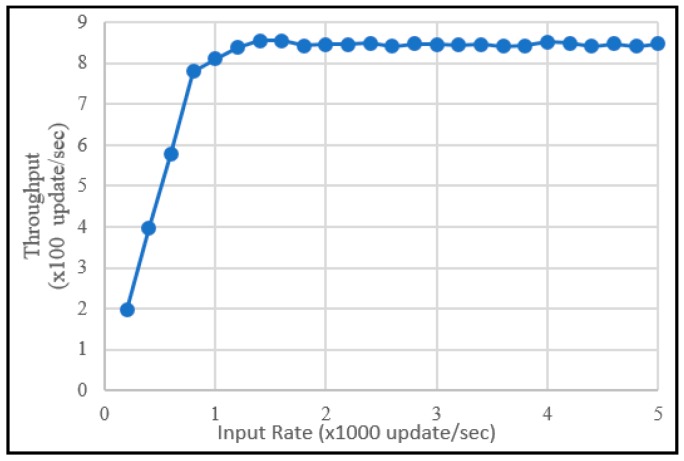
Throughput vs. input rate.

**Figure 29:**
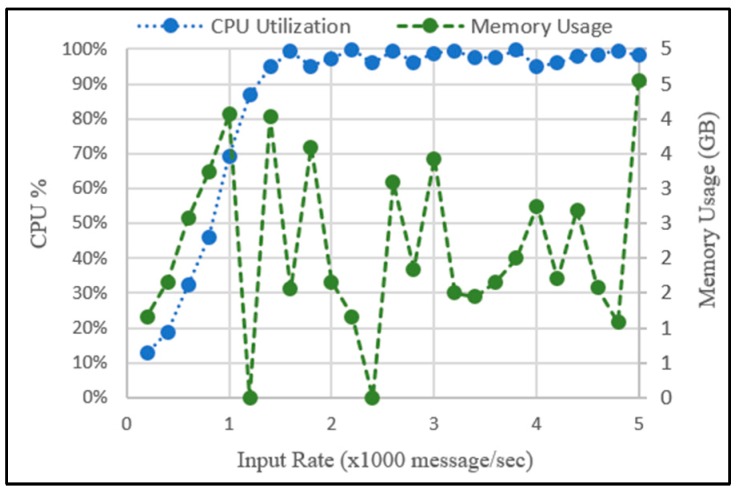
Resource utilization.

**Figure 30:**
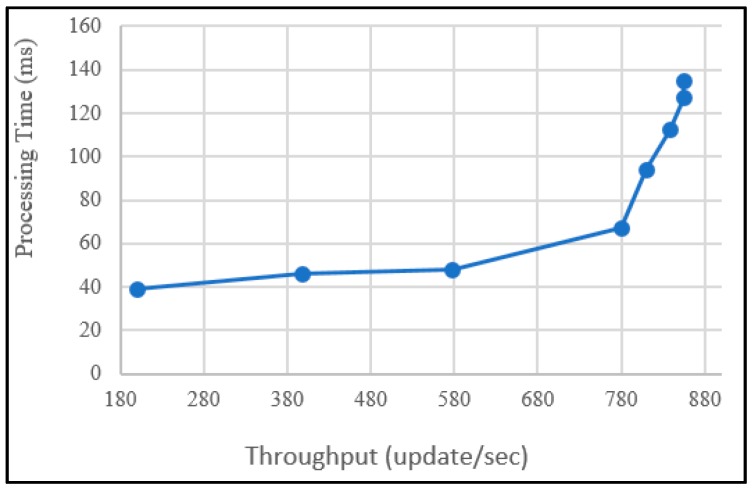
Processing time vs. throughput.

During the experiment, we were increasing the input rate from 200 updates per second to 5000 updates per second, while keeping the number of subscriptions equal to 10. Similar to experiment 1, we ran each test for 10 min and calculated the average throughput. The result of this experiment is depicted in Figure 28, Figure 29 and Figure 30.

Figure 28 shows the impact of increasing the input rate on throughput. As the graph shows, while the input rate increased from 200 to 1000, the throughput grows linearly with almost direct ratio from 196 updates/sec to 878 updates/s. From that moment until the end of the experiment, the throughput remained on the same level as the CPU utilisation (Figure 29) reached its maximum. During this period, as the input rate became higher than the throughput, the messages were queued.

To demonstrate the effect of throughput on the processing time of an update, we plotted Figure 30. This graph shows gradual linear growth of the processing time from 40 ms to slightly more than 67 ms until the throughput reached 780 updates per second, which is the CPU saturation point. Then, updates started queueing and the processing time dramatically increased.

**Figure 31:**
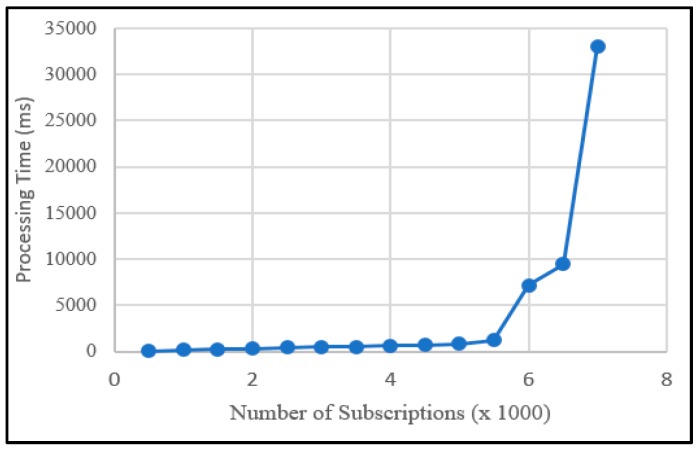
Processing time vs. number of subscriptions.

**Figure 32:**
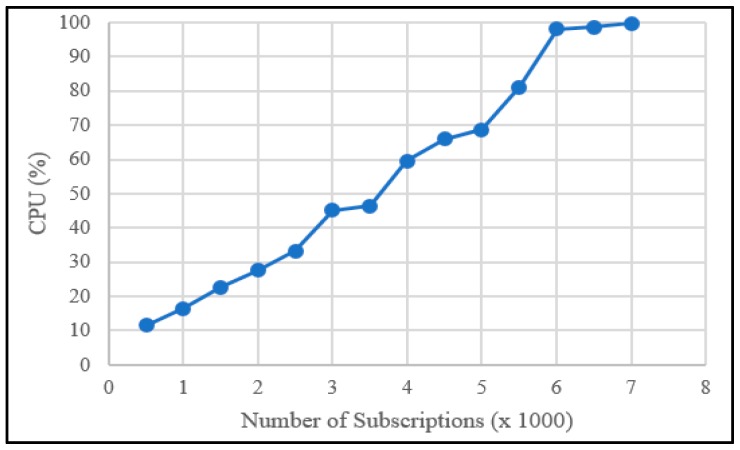
CPU utilisation vs. number of subscriptions.

The second experiment analyses how the number of subscriptions affects the context update processing time. We varied the number of subscriptions from 500 to 7000 while the input rate was equal to 100 updates per second. The result of this experiment is presented in Figure 31 and Figure 32. As it can be seen, the processing time increased gradually from 84 ms to 1239 ms, while the number of subscriptions increased from 500 to 5500. After that point, as the CPU utilisation reaches its maximum, the processing time increased dramatically.

In both sets of experiments, we demonstrated how the CoaaS platform could handle increasing load with high performance. We observed a drop in performance when the incoming load became too high. The result of our analysis shows and demonstrates that the drop in performance was caused by the resource limitation as we conducted our study with one server instance. However, all the components used in the system design were stateless and could be easily scaled out to several instances to provide near real-time performance for IoT scale applications.

## 8. Conclusions

In this paper, we presented a novel Context Definition and Query Language (CDQL). The CDQL language is being considered by ETSI CIM group [18] as complementary to its current proposed draft of NGSI-LD (https://www.etsi.org/news-events/news/1300-2018-04-news-etsi-isg-cim-group-releases-first-specification-for-context-exchange-in-smart-cities) especially in addressing high-level context- and situation-awareness. The CDQL aims to define and represent context entities and context requests for IoT applications, services, and systems. CDQL consists of two main parts namely: Context Definition Model, which describes high-level context and situations, and Context Query Language (CQL), which is a powerful and flexible query language to express contextual information requirements without considering details of the underlying data structure. CQL supports both pull- and push-based queries. One of the main features of this language is its ability to support and represent contextual functions, namely situation (high-level context) and aggregation functions. The proposed language demonstrated its expressiveness, and rich functionality in EU Horizon-2020 project biotope (www.biotope-project.eu) use cases. We also evaluated the proposed CDQL in terms of performance and scalability by conducting several experiments. These experiments showed the proposed language can be utilised for large-scale IoT applications in different domains. The future work will include further functionality extensions, applying CDQL to more use cases, as well as developing a two-way gateway between CDQL and NGSI-LD.

## Figures and Tables

**Table 1:** Evaluation of Existing CQLs.

Title	CQL Type	Requirements					
		*#1*	*#2*	*#3*	*#4*	*#5*	*#6*
Contory [38]	SQL-based	✖	✖	✔	✖	✖	✔
CML [39]	SQL-based	↘	✖	✖	↘	↘	✔
PerLa [42]	SQL-based	↗	✖	✔	↘	↗	↘
NGSI-9/10 [43]	API-based	✖	↗	✔	↘	✔	↗
SPARQL [44]	RDF-based	✔	↗	✖	✖	✖	✖
MUSIC-CQL [45]	RDF-based	✖	✔	✔	↗	↗	↗
SOCAM [8]	RDF-based	✖	✔	✔	↘	↗	↘
Nexus [47]	XML-based	↗	↘	✔	✖	✔	✔
MobiLife [46]	XML-based	↘	✔	✔	↘	✖	↗
ContextML [56]	XML-based	↗	✔	✔	↘	✖	↗

✔ = supported, ✖ = unsupported, ↗ = most important features supported, ↘ = very limited support.

## Supplementary Materials

The following are available online at https://www.mdpi.com/1424-8220/19/6/1478/s1.
