# Supplementary material for: Context Definition and Query Language: Conceptual Specification, Implementation, and Evaluation
Source: Sensors (Basel). 2019 Mar 26;19(6):1478. doi: 10.3390/s19061478 (PMC6470624; doi:10.3390/s19061478)
Supplement: Supplementary file 1 [file sensors-19-01478-s001.pdf]

Supplementary Material

Context Definition and Query Language: Conceptual Specification, Implementation, and Evaluation

Alireza Hassani [1,*], Alexey Medvedev [1], Pari Delir Haghighi [1], Sea Ling [1], Arkady Zaslavsky [2] and Prem Prakash Jayaraman [3]

1 Faculty of Information Technology, Monash University, Melbourne 3145, Australia; alexey.medvedev@monash.edu (A.M.); pari.delir.haghighi@monash.edu (P.D.H.); chris.ling@monash.edu (S.L.)
2 School of Information Technology, Deakin University, Geelong, Vic 3216, Australia; arkady.zaslavsky@deakin.edu.au
3 Department of Computer Science and Software Engineering, Swinburne University of Technology, Melbourne 3122, Australia; pjayaraman@swin.edu.au
* Correspondence: ali.hassani@monash.edu; Tel.: +61-3-9903-2556

A. CQL EBNF

```
CDQL     ::= DML_STATEMENT

           | DDL_STATMENT


DML_STATEMENT ::= PREFIX SELECT WHEN? DEFINE SET?


PREFIX   ::= 'prefix' PREFIX_ID ':' URI ( ',' 'prefix' PREFIX_ID ':' URI )*


SELECT   ::= 'select' '(' ( CONTEXT-ATTRIBUTE | CONTEXT-ENTITY | FUNCTION-CALL )
( 'as' IDENTIFIER )? ( ',' ( CONTEXT-ATTRIBUTE | CONTEXT-ENTITY | FUNCTION-CALL
) ( 'as' IDENTIFIER )? )* ')'


CONTEXT-ATTRIBUTE

         ::= CONTEXT-ENTITY-ID ( '.' IDENTIFIER )+


FUNCTION-CALL

         ::= ( PACKAGE-TITLE '::' )? FUNCTION-NAME '(' ( CONTEXT-ATTRIBUTE |
CONTEXT-ENTITY-ID | FUNCTION-CALL ) ( ',' ( CONTEXT-ATTRIBUTE | CONTEXT-ENTITY-
ID | FUNCTION-CALL ) )* ')' ( '.' IDENTIFIER )*


DEFINE   ::= 'define' 'entity' CONTEXT-ENTITY-ID 'is form' Prefix_ID ':'
Entity_title ( 'where' CONDITION )? SORT-BY? ( ',' 'entity' CONTEXT-ENTITY-ID
'is form' Prefix_ID ':' Entity_title ( 'where' CONDITION )? SORT-BY? )*
```

CONDITION

::= (CONTEXT-VALUE | CONTEXT-ATTRIBUTE | FUNCTION-CALL) (Comparision-Operator | Logical-Operator) (CONTEXT-VALUE | CONTEXT-ATTRIBUTE | FUNCTION-CALL)?

| (CONDITION ('and' | 'or') | 'not') CONDITION

| '(' CONDITION ')'

SORT-BY ::= 'sort by' (CONTEXT-ATTRIBUTE | FUNCTION-CALL | ARITHMETIC-EXPRESSION) (',' (CONTEXT-ATTRIBUTE | FUNCTION-CALL | ARITHMETIC-EXPRESSION))* ('asc' | 'desc')?

WHEN ::= ('when' HIGH-LEVEL-SITUATION | 'every' duration) ('until' (date '/' ?) (date | duration | number 'occurrences'))?

duration ::= 'P' (digit+ 'Y')? (digit+ 'M')? (digit+ 'D')? ('T' (digit+ 'H')? (digit+ 'M')? (digitd+ 'S')?)?

SET ::= 'set' ('callback' ':' '{' 'method' ':' METHOD ',' 'body' ':' string | 'meta' ':' '{' META-DATA-KEY ':' CONTEXT-VALUE (',' META-DATA-KEY ':' CONTEXT-VALUE)* | 'output' ':' '{' OUTPUT-CONFIG) '}'

OUTPUT-CONFIG

::= 'structure' ':' STRUCTURE (',' 'vocabulary' ':' '{' CONTEXT-ENTITY-ID ':' PREFIX_ID ':' Entity_title (',' CONTEXT-ENTITY-ID ':' PREFIX_ID ':' Entity_title)* '}')?

DDL_STATMENT

::= CREATE-FUNCTION

| 'create' 'package' PACKAGE-NAME

| 'alter' 'package' PACKAGE-NAME 'set' 'title' PACKAGE-TITLE

| 'drop' 'function' (PACKAGE-TITLE '::')? FUNCTION-NAME

CREATE-FUNCTION

::= PREFIX 'create function' FUNCTION-NAME 'is on' (Prefix_ID ':' Entity_title | Data_Type) 'as' Identifier (',' (Prefix_ID ':' Entity_title |

```
Data_Type ) 'as' Identifier )* ( SITUATION-FUNCTION | AGGREGATION-FUNCTION ) ('set package' PACKAGE-TITLE)?

AGGREGATION-FUNCTION

         ::= ( 'post' | 'get' ) ( 'http' | 'https' ) '://' host ( ':' port )? ( '/' ( normal_path | path_param ) )? ( '?' ( normal_query | query_param ) )?

SITUATION-FUNCTION

         ::= CST-SITUATION

           | HIGH-LEVEL-SITUATION

CST-SITUATION

         ::= SITUATION-NAME ':' '{' CONTEXT-ATTRIBUTE ':' CST-ATTRIBUTE-DEFINITION ( ',' CONTEXT-ATTRIBUTE ':' CST-ATTRIBUTE-DEFINITION )* '}' ( ',' SITUATION-NAME ':' '{' CONTEXT-ATTRIBUTE ':' CST-ATTRIBUTE-DEFINITION ( ',' CONTEXT-ATTRIBUTE ':' CST-ATTRIBUTE-DEFINITION )* '}' )*

CST-ATTRIBUTE-DEFINITION

         ::= '{' 'ranges' ':' '[' '{' 'value' ':' ( '[' | '(' ) number ';' number ( ')' | ']' ) ',' 'belief' ':' number '}' ( ',' '{' 'value' ':' ( '[' | '(' ) number ';' number ( ')' | ']' ) ',' 'belief' ':' number '}' )* ']' ',' 'weight' ':' number '}' '}'

HIGH-LEVEL-SITUATION

         ::= ( CONTEXT-VALUE | CONTEXT-ATTRIBUTE | FUNCTION-CALL ) ( Comparision-Operator | Logical-Operator ) ( CONTEXT-VALUE | CONTEXT-ATTRIBUTE | FUNCTION-CALL )?

           | ( HIGH-LEVEL-SITUATION ( Logical-Operator | Allens-Algerbar-OP ) | 'not' ) HIGH-LEVEL-SITUATION

           | '(' HIGH-LEVEL-SITUATION ')'
```

B. CDQL Built-in Functions

Function Title	Details
Max(argument, [window])	Return the maximum value of a given argument. If the window is provided, the value will be calculated during the provided window.
Min(argument, [window])	Return the minimum value of a given argument. If the window is provided, the value will be calculated during the provided window.

`Sum(argument, [window])`	Return the total sum of a given argument. If the window is provided, the value will be calculated during the provided window.
`Average(argument, [window])`	Return the average of a given argument. If the window is provided, the value will be calculated during the provided window.
`SD(Ca, [window])`	Returns the standard deviation of given argument during the provided window.
`Count(argument, [window])`	Return the number of times value of a given argument has been updated. If the window is provided, the value will be calculated during the provided window.
`Increased(argument, window)`	Returns true when the value of a given attribute increased during the provided window.
`Decreased(argument, window)`	Returns true when the value of a given attribute decreased during the provided window.
`isValid(argument, window)`	Returns true when the value of a given attribute is unchanged during the provided window.
`change(argument, [value],[ window])`	Returns true when the value of a given attributes changes. If the value is provided, returns true only the value of the given attribute change to the provided value. In all the other cases returns False.<br>If the window is provided, the value will be calculated during the provided window.
`Distance(origin, destination,[transport_type])`	Returns a JSON result which contains the Euclidean distance between the origin and destination. If the transport_type is provided, returns the travel distance and time for a given origin and destination, based on the recommended route between start and end points considering the travel mode. The following travel modes are supported: driving, walking, bicycling, and transit.
`Intersect(Geo-shape*, Geo-shape*)`	Allows you to compare two geospatial types to see if they intersect or overlap each other.
`SpatioTemporalIntersect(Route*, Route*)`	Returns true if the provided routes have an intersection considering both location and time.
Within(`Geo-shape*, Geo-shape*`)	Returns true if the first geo-shape is inside the second Geo-shape.

* All the Geo-shape can be represented either by GeoJSON format or Well-known Text markup language.
